# Bacterial Dispersers along Preferential Flow Paths of a Clay Till Depth Profile

**DOI:** 10.1128/AEM.02658-18

**Published:** 2019-03-06

**Authors:** U. S. Krüger, A. Dechesne, F. Bak, N. Badawi, O. Nybroe, J. Aamand

**Affiliations:** a Geological Survey of Denmark and Greenland, Copenhagen, Denmark; b University of Copenhagen, Department of Plant and Environmental Sciences, Copenhagen, Denmark; c Technical University of Denmark, Department of Environmental Engineering, Lyngby, Denmark; University of Illinois at Urbana-Champaign

**Keywords:** community motility, liquid film, preferential flow paths, soil, succession

## Abstract

This study assessed the dispersal of five bacterial communities from contrasting compartments along a fractured clay till depth profile comprising plow layer soil, preferential flow paths (biopores and the tectonic fractures below), and matrix sediments, down to 350 cm below the surface. A recently developed expansion of the porous surface model (PSM) was used to capture bacterial communities dispersing under controlled hydration conditions on a soil-like surface. All five communities contained bacteria capable of active dispersal under relatively low hydration conditions (−3.1 kPa). Further testing of the plow layer community revealed active dispersal even at matric potentials of −6.3 to −8.4 kPa, previously thought to be too dry for dispersal on the PSM. Using 16S rRNA gene amplicon sequencing, the dispersing communities were found to be less diverse than their corresponding total communities. The dominant dispersers in most compartments belonged to the genus *Pseudomonas* and, in the plow layer soil, to *Rahnella* as well. An exception to this was the dispersing community in the matrix at 350 cm below the surface, which was dominated by *Pantoea*. Hydrologically connected compartments shared proportionally more dispersing than nondispersing amplicon sequence variants (ASVs), suggesting that active dispersal is important for colonizing these compartments. These results highlight the importance of including soil profile heterogeneity when assessing the role of active dispersal and contribute to discerning the importance of active dispersal in the soil environment.

**IMPORTANCE** The ability to disperse is considered essential for soil bacteria colonization and survival, yet very little is known about the dispersal ability of communities from different heterogeneous soil compartments. Important factors for dispersal are the thickness and connectivity of the liquid film between soil particles. The present results from a fractured clay till depth profile suggest that dispersal ability is common in various soil compartments and that most are dominated by a few dispersing taxa. Importantly, an increase in shared dispersers among the preferential flow paths of the clay till suggests that active dispersal plays a role in the successful colonization of these habitats.

## Abstract (precis)

The ability to disperse is considered essential for soil bacteria colonization and survival, yet very little is known about the dispersal ability of communities from different heterogeneous soil compartments. Important factors for dispersal are the thickness and connectivity of the liquid film between soil particles. The present results from a fractured clay till depth profile suggest that dispersal ability is common in various soil compartments and that most are dominated by a few dispersing taxa. Importantly, an increase in shared dispersers among the preferential flow paths of the clay till suggests that active dispersal plays a role in the successful colonization of these habitats.

## INTRODUCTION

Bacterial dispersal in soil has long been considered an important topic of study for microbiologists in various contexts, such as bioremediation, ecology, plant protection, and community dynamics (1–5). While these studies provide essential insights, they are mostly based on observations from pure culture studies, leaving much still unknown about dispersal in natural soil communities.

Bacteria disperse either passively, e.g., by random movement (Brownian motion), transport on plant roots, or with water flow, or actively, which requires energy, often using dedicated cellular appendages such as flagella (2, 6–8). In recent studies, there is also an increasing awareness of the potential for cooperative dispersal strategies, such as cargo transport of nonmotile bacteria by motile bacterial swarms (3, 9) or interkingdom cooperation with dispersal facilitated by fungi or amoeba (10–13). However, methods for assessing dispersal ability of complex bacterial communities under conditions relevant to soil have only lately become available (14, 15).

Bacteria are aquatic organisms by nature and require an aquatic environment for their life functions (16). In soil, water is also crucial to dispersal, because bacterial cells generally need to be fully immersed in liquid to move (2, 17). As a consequence, bacterial dispersal in soil is limited to microhabitats that are interconnected by water pathways, such as the liquid films between soil particles (2, 7). This makes soil water a key factor in bacterial dispersal and consequently in bacterial survival and community diversity. Indeed, connectivity, or more accurately, the lack of it, is important for maintaining the huge microbial diversity found in the heterogeneous soil environment (2, 18–21). Connectivity in soil can be considered at different scales, from a microscale at which a single bacterium operates to a macroscale, e.g., an agricultural field.

At the macroscale, the flow of water in well-structured soils is mainly restricted to preferential flow paths, closely connecting some parts of the soil profile while leaving others isolated (7, 22, 23). A “text book” example of connectivity at the macroscale is agricultural clay tills, where most of the water, primarily from rainfall, moves from the plow layer through preferential flow paths toward groundwater reservoirs. These preferential flow paths comprise a complex system of biopores (mainly earthworm burrows and plant root channels) that are connected to tectonic fractures in deeper layers (22, 24, 25).

In clay till, preferential flow paths are fairly well characterized from a geological perspective (24–27), particularly as a result of their potential importance in the leaching of pesticides and other contaminants to groundwater (28). However, from a microbial perspective, much is still unclear. Soils separated by a few meters may have very different community structures (2, 6). Indeed, communities separated by as little as a few millimeters can vary in composition, activity, and function, e.g., the potential for degradation of pesticides (6, 20, 29). This spatial influence on bacterial communities may be pronounced in clay tills, where the soil profile can be viewed as consisting of spatially isolated compartments, as well as in fracture surfaces and matrix sediment, for example, which provide bacterial habitats with vastly different physical and chemical compositions (24–27, 30). These various conditions can select for different bacteria, leading to differences in community compositions (31). Dispersal has the potential to redistribute bacteria and spatially homogenize the community composition. While preferential flow paths can be a major route for the passive transport of bacteria through soil (7, 16, 32, 33), the contribution of active dispersal to community assembly processes in soil and sediments has not been explored.

Some of the most important factors potentially limiting active dispersal in soil and deeper sediments are fluctuating matric potentials and the subsequent loss of connectivity at the microscale, as has been shown in pure culture studies that have highlighted the negative effect of increasingly thin liquid films on flagellum-mediated dispersal (18, 34, 35). However, these limitations might not apply to the same extent to the dispersal of environmental communities. Using the novel and extended porous surface model (PSM) method to study bacterial dispersal under controlled hydration conditions on a soil-like surface, Krüger et al. found that part of environmental communities were able to disperse even under conditions previously thought too dry for dispersal (14, 18, 35). According to their observations, rapid dispersal was possible even at a matric potential of −4.2 kPa, but the community response to increasingly negative matric potentials, and thus decreased liquid film thickness and connectivity, has not been investigated beyond that point.

In the present study, the aim was to assess the dispersal potential of bacterial communities from five compartments of a well-defined agricultural soil profile covering the plow layer, deeper preferential flow paths (biopores and tectonic fractures), and adjacent matrix sediments. It was hypothesized that (i) a subcommunity of efficient dispersers is present in each compartment, and (ii) these bacteria are frequently shared between hydraulically connected compartments. Furthermore, the effect of low matric potential, and thus, a thin liquid film, on dispersal of a plow layer soil bacterial community was studied and it was hypothesized that (iii) only a fraction of the motile community is able to disperse under low hydration conditions.

## RESULTS

### Bacterial communities recovered from the soil profile.

**FIG 1:**
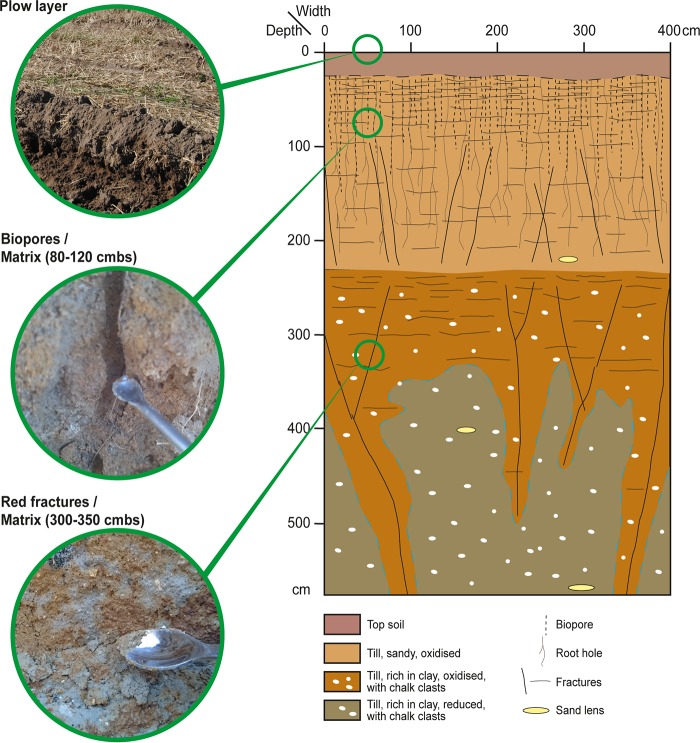
Schematic illustration of the soil profile with highlighted sampling points. Plow layer samples were obtained from 20 cmbs (cm below surface). Biopores and matrix sediment samples were from 80 to 120 cmbs, and red fractures and matrix sediment were sampled from 300 to 350 cmbs. The illustration is adapted with permission from a report from the Danish Pesticide Leaching Assessment Programme (PLAP; http://pesticidvarsling.dk).

**FIG 2:**
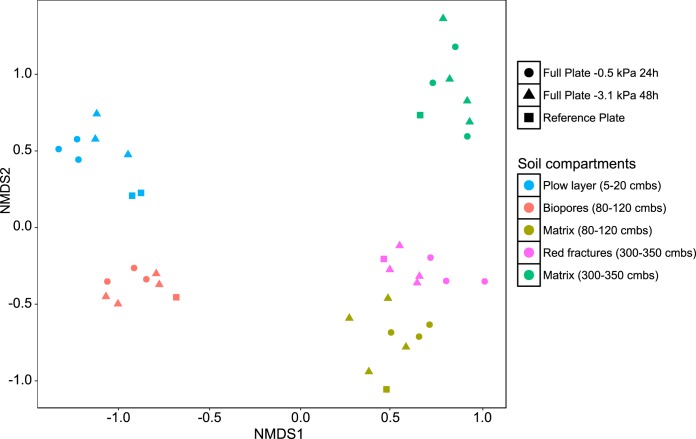
NMDS plot of the compositions of the total communities derived from five compartments of a well-defined soil profile. Stress, 0.13. Bray-Curtis dissimilarities calculated from 16S rRNA genes. The total communities were tested at two matric potentials in the PSM experiments, −0.5 kPa and −3.1 kPa, and recovered on full agar plates (full plate). The motility-restricted controls (reference plate) are marked with squares. Replicates are depicted as separate dots.

This study assessed the dispersal of five bacterial communities extracted from five different compartments of a well-defined clay till depth profile (Fig. 1). A newly developed method, the extended porous surface model (PSM), was used, in which agar plate imprints are used to reveal the spatial spreading of bacterial communities on a rough hydrated surface resembling soil. This method allows for the recovery and characterization of both the dispersing bacteria and the total community, recovered by pressing a hollowed-out agar plate and a “full” agar plate, respectively, onto the PSM surface. The total bacterial communities from the five soil and sediment compartments clearly separated into five clusters on the nonmetric multidimensional scaling (NMDS) plot of the community composition from 16S rRNA amplicon sequence data. This was confirmed by permutational multivariate analysis of variance (PERMANOVA) analysis on Bray-Curtis dissimilarities, where 56% of the variance was explained by soil compartment (*P* < 0.001) (Fig. 2). Heatmaps and Venn diagrams of the amplicon sequence variants (ASVs) (36) of the total communities also illustrate the different community compositions (see Fig. S1 to S6 in the supplemental material). Comparisons between the original soil community, the inoculum (Nycodenz extractions), and the cultivable communities on the full plates and reference plates (inoculum placed directly on agar plate) confirm the expected cultivation bias (see Fig. S7 and S8). Yet, in general, the total cultivable communities retained a high level of diversity, with representatives of 109 unique genera (belonging to 5 different phyla), across all compartments plus 161 ASVs that were not identifiable at the genus level (see Table S1). Overall, 27.7% and 8.7% of the genera in abundances of >0.1% in the biopores and plow layer soil communities, respectively, were recovered on the full plates pressed onto the PSM incubated for 48 h at −3.1 kPa. Similar values were observed for the −0.5-kPa/24-h samples (see Table S2). Additionally, the ASVs found on the full plates represented 10% and 1% of the original community from the biopores and plow layer soil, respectively (see Table S3). This signifies that the applied method was able to recover a substantial part of the diversity present in the original soil communities.

The genera *Pseudomonas*, *Flavobacterium*, and *Pedobacter* dominated the total communities of the plow layer, biopores, and matrix at 80 to 120 cm below surface (cmbs) (Fig. S1 to S3), while in the fracture community at 300 to 350 cmbs, *Flavobacterium* was replaced by *Arthrobacter* (Fig. S4). The community from the deep matrix sediment at 300 to 350 cmbs was dominated by the genus *Pantoea*, followed by *Pseudomonas*, *Chryseobacterium*, and *Stenotrophomonas* (Fig. S5). The moisture conditions on the PSM model (−0.5 and −3.1 kPa) had only a minor influence on the total bacterial community composition, contributing just 7% of the variation in the PERMANOVA analysis of Bray-Curtis dissimilarities (*P* < 0.001) (Fig. 2 and Fig. S1 to S5). In conclusion, the soil communities recovered from the PSM were distinctly different, although they shared some dominant genera.

### Community dispersal potential and identity of major dispersers.

**FIG 3:**
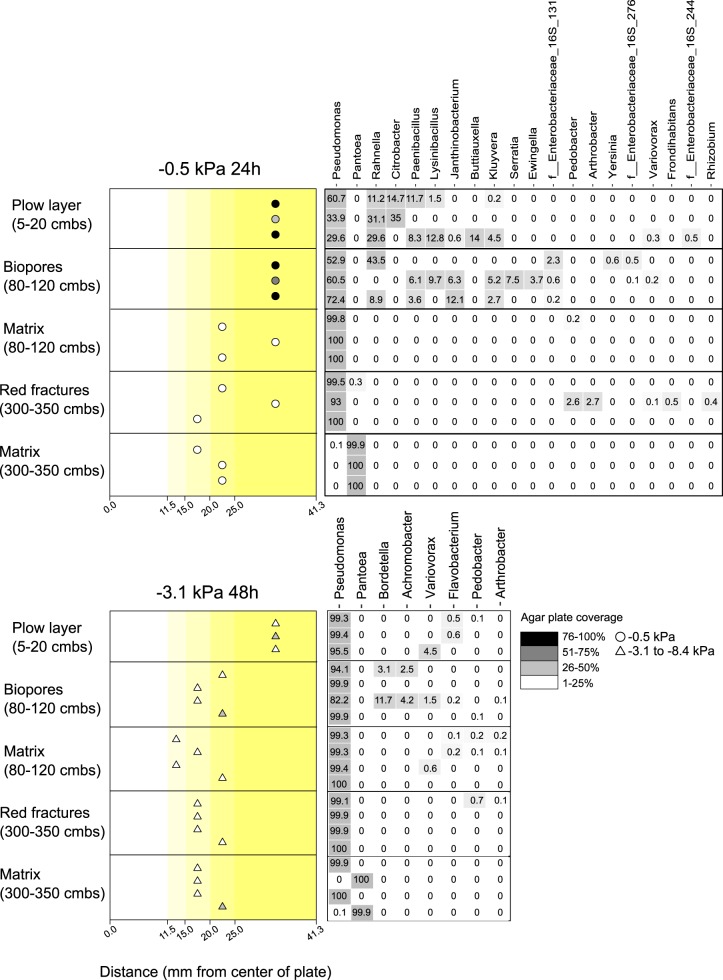
Dispersal and composition of communities derived from five compartments of a well-defined soil profile and incubated at matric potential −0.5 kPa for 24 h (A) and −3.1 kPa for 48 h (B). (Left) Symbol shading depicts bacterial coverage of the pressed agar plate, giving an indication of the extent of colonization. The distances shown are ranges, e.g., colonies were observed on the agar ring at distances of between 11.5 and 15 mm from the inoculation point at the center. (Right) Heatmap of the relative abundance of the most dominant genera among the dispersing bacteria across five soil communities. The replicates are depicted as separate dots, and replication numbers varied from three to four.

Rapid dispersal of bacteria was observed for all soil and sediment communities under wet conditions (−0.5 kPa). Except for the plow layer community, there was a clear tendency toward slower dispersal and lower surface coverage scores under dry conditions (−3.1 kPa) compared to that under wet conditions, indicating dispersal limitation under dry conditions (Fig. 3).

Using 16S rRNA gene amplicon sequencing, the dispersing bacteria from the extracted soil and sediment communities were identified. The compositions of these dispersing communities were then compared to those of the total bacterial communities. Both the Shannon diversity and Faith’s phylogenetic diversity indices showed that the total communities were more diverse than the dispersed communities and that the dispersers had a narrow phylogenetic diversity (see Fig. S9 and S10). The Shannon diversity index also revealed a lower diversity under dry conditions (−3.1 kPa) compared to that under wet conditions for all dispersed communities, except for the matrix sediment at 80 to 120 cmbs, where a high variation between replicates was seen. Dispersing bacteria predominantly belonged to the genus *Pseudomonas* in all but one community at −0.5 kPa. Additionally, under these wet conditions, the plow layer and the biopore dispersers shared a high relative abundance of *Rahnella*, *Paenibacillus*, *Lysinibacillus*, and *Kluyvera* (Fig. 3). Under dry conditions, *Pseudomonas* almost completely dominated the dispersed communities, except for the matrix soil at 300 to 350 cmbs. Here *Pantoea* was dominant at −0.5 kPa, while at −3.1 kPa, *Pantoea* and *Pseudomonas* were represented equally. In general, the dominant disperser genera were also represented in the total community, but they were greatly enriched in the disperser communities.

On an NMDS plot (see Fig. S11 and S12), the dispersed communities separated from the total communities, as confirmed by PERMANOVA analysis on Bray-Curtis dissimilarities, explaining 9% and 11% of the variance for −0.5 and −3.1 kPa (all *P* < 0.001), respectively. However, the strongest effect was still attributed to the compartment type, explaining 47% and 28% of the variance under wet and dry conditions, respectively. Additionally, there was a significant, but moderate, interaction between dispersed/total community and soil compartment (14%, *P* < 0.001, for −0.5 kPa/24 h and 15%, *P* < 0.001, for −3.1 kPa/48 h, respectively). Significant differences in homogeneity between the dispersed and total communities were found using Betadisperser followed by analyses of variance (ANOVA), which tested whether the dispersion of a group from its median was different from the dispersion of other groups (*F* = 5.9, *P* < 0.05, for −0.5 kPa/24 h and *F*= 7.1, *P* < 0.01 for −3.1 kPa/48 h). Hence, the dispersed communities had a significantly greater variation than the total communities, indicating a stochastic element in the identity of the bacterial dispersers.

### Connectivity of dispersing communities from preferential flow paths and matrix.

**FIG 4:**
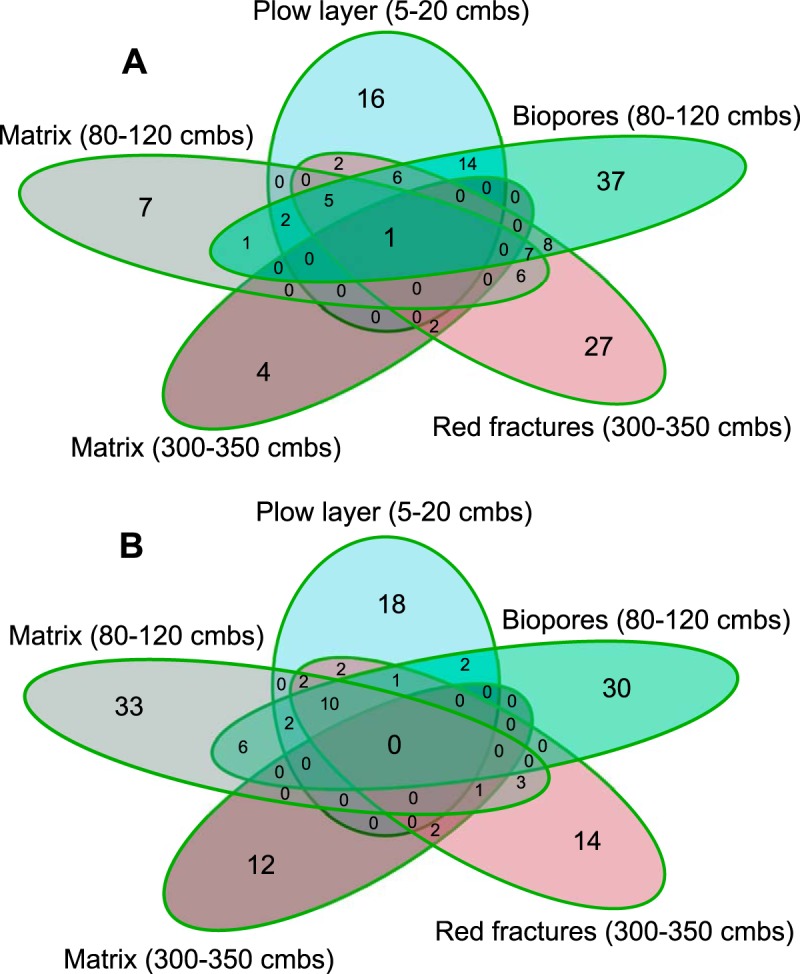
Venn diagrams depicting the shared and unique ASVs between the dispersed communities from five compartments of a well-defined soil profile. (A) Communities exposed to −0.5 kPa for 24 h, with a total of 145 unique ASVs. (B) Communities exposed to −3.1 kPa for 48 h, with a total of 138 unique ASVs.

A closer look at the ASVs in the dispersed communities (Fig. 4A and B and Tables S4 and S5) revealed that many ASVs were shared between the plow layer, biopores, and fracture communities. The number of shared ASVs was generally much higher under wet conditions than under dry conditions. The most common genus among the shared dispersers between the three communities of the plow layer, biopores, and fractures under both hydration conditions was *Pseudomonas* (10 shared ASVs at −0.5 kPa and −3.1 kPa), but *Buttiauxella* was also represented (1 shared ASV at −0.5 kPa and −3.1 kPa). The one ASV shared between all compartments under wet conditions belonged to the genus *Pseudomonas*.

A comparison of the percentages of shared ASVs between dispersing and nondispersing bacteria from the preferential flow paths also revealed a very clear picture where the dispersers were shared more than nondispersers (Table 1). It should be noted that what are referred to as “nondispersers” are actually the ASVs in the total communities minus the ASVs observed among the dispersers. This group may therefore also contain some slow dispersers, which were not quick enough to be detected among the dispersers. The proportion of shared dispersers was significantly higher than the proportion of shared nondispersers in the preferential flow paths for both wet and dry conditions, although under dry conditions, this was only significant for the shared communities between the plow layer and fractures. In contrast to the greater sharing of dispersers along the preferential flow paths, there was no significant preferential sharing of dispersers between the preferential flow paths and the adjacent matrix sediments. Indeed, in some cases, there was even greater sharing of nondispersing ASVs between these compartments, as was also the case for vertical sharing between the 80- to 120-cmbs matrix and 300- to 350-cmbs matrix sediment communities.

**TABLE 1:** Shared dispersing and nondispersing ASVs between communities derived from five compartments of a well-defined soil profile

Comparison	Matric potential (kPa)	% of shared dispersers^*a*^	% of shared nondispersers^*b*^	*P* value^*c*^
Preferential flow paths				
Plow layer vs biopores	−0.5	28.9	12.2	0.001
	−3.1	20.0	15.0	0.2892
Biopores vs fractures	−0.5	22.9	5.6	0.001
	−3.1	14.7	7.6	0.1042
Plow layer vs fractures	−0.5	14.6	3.8	0.01
	−3.1	26.3	6.9	0.001
Preferential flow path vs matrix				
Biopores vs matrix at 80–120 cmbs	−0.5	17.0	13.6	0.5636
	−3.1	20.0	12.6	0.1093
Fractures vs matrix at 300–350 cmbs	−0.5	4.4	13.3	0.0826
	−3.1	6.4	17.8	0.07794
Matrix vs matrix				
Matrix 80–120 cmbs vs matrix 300–350 cmbs	−0.5	2.9	10.1	0.2712
	−3.1	1.4	11.9	0.05

a Dispersed bacteria recovered the furthest from the inoculation point (at least 11.5 mm).

b Nondispersing bacteria were calculated by subtracting the unique ASVs observed in the dispersed community from the ASVs observed in the total community.

c *P* values of Fisher’s exact tests.

### Response to increasingly negative matric potentials.

**FIG 5:**
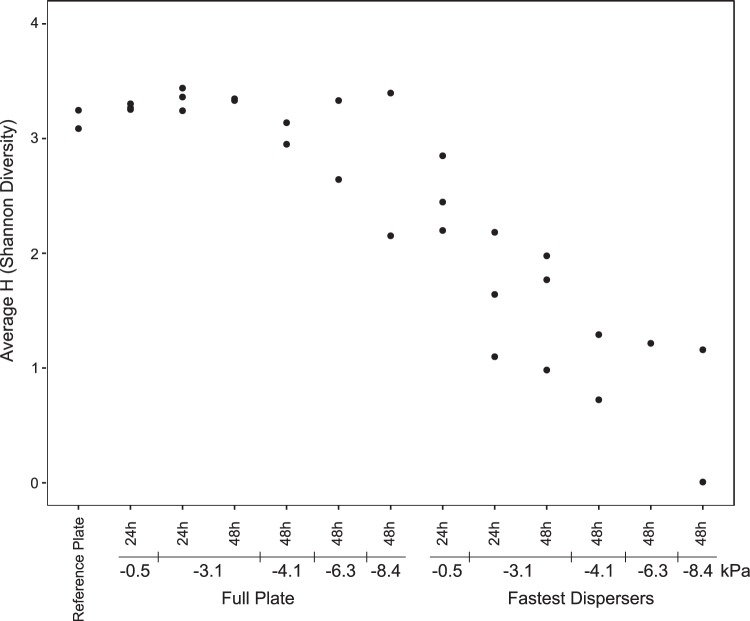
Estimates of alpha-diversity (Shannon diversity index) for communities derived from plow layer soil samples and incubated at a range of negative matric potentials (−0.5 to −8.4 kPa) for 24 h or 48 h. For each replicate PSM, the total community recovered from the full agar plate (full plate) and the dispersed community are presented. A motility-restricted control (reference plate) is also included. Replicates are depicted as separate dots.

**FIG 6:**
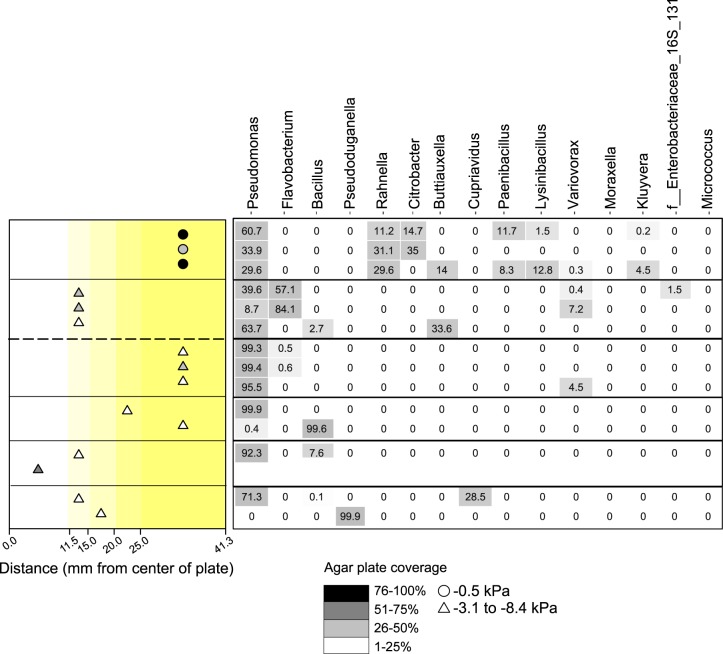
Dispersal and composition of a community extracted from plow layer soil and incubated at matric potentials from −0.5 kPa to −8.4 kPa for 24 h or 48 h. (Left) Symbol shading depicts bacterial coverage of the pressed agar plate, giving an indication of the extent of colonization. The distances shown are ranges, e.g., colonies were observed on the agar ring at distances of between 11.5 and 15 mm from the inoculation point at the center. (Right) Heatmap of the relative abundance of the most dominant genera among the dispersers. Replication numbers varied from two to three.

To test the effect of increasingly negative matric potentials on the dispersal ability of a soil community, the community extracted from the plow layer soil was exposed to matric potentials from −0.5 to −8.4 kPa. Interestingly, dispersal was seen even under the driest conditions, but the Shannon diversity index of the dispersing community decreased as the conditions became dryer (Fig. 5), with only very few genera present under dry conditions (Fig. 6 and Fig. S13). Furthermore, Faith’s diversity index extended the previous results, showing that the narrow phylogenetic distribution of the dispersed communities became narrower under even dryer conditions (see Fig. S14).

In general, dispersal was increasingly restricted at matric potentials of −4.1 kPa and lower (Fig. 6), with no replicates dispersing beyond the 15- to 20-mm section for matric potentials of −6.1 and −8.4 kPa. An element of randomness was involved in the identity of the dispersers at −4.1 to −8.4 kPa, with *Pseudomonas* still being prominent, but in some replicates, *Cupriavidus*, *Bacillus*, or *Pseudoduganella* were also dominant dispersers.

The element of randomness at decreased matric potentials was also supported by visual observations of the colonization patterns on the surfaces of the agar plates (see Fig. S15). While at −0.5 and −3.1 kPa, the patterns were characterized by a relatively uniform spread of bacteria from the inoculation point to the edge of the plate, at −6.3 and −8.4 kPa, dispersal was limited to a few corridors.

## DISCUSSION

### Dispersal potential and disperser identity in communities from fractured clay till.

While the role of passive transport is well established (2, 32, 33), the importance of active dispersal in soil has been debated for many years (2, 3, 7, 17). Until recently, there was no experimental platform to screen for active dispersal at the community level (14, 15). The present study assessed the dispersal of five bacterial communities from matrix sediments and preferential flow paths of a clayey till.

The soil and sediment compartments studied here harbored different bacterial communities, reflecting the very heterogeneous nature of clay till profiles and confirming the existence of distinct compartments (30, 31). Dispersing bacteria were found in the plow layer and in all deeper sediments, pointing to the importance of active dispersal in these environments, though these dispersers were not dominant in the total communities (14, 15). *Pseudomonas* was the dominant disperser in the top soil, in agreement with results from the few comparable studies available (14, 15). Members of the *Pseudomonadacea*e family have also been found to be early colonizers of plant litter in an agricultural field, also suggesting that *Pseudomonas* is a key disperser in the soil environment (37). In the literature, pseudomonads are known to be efficient dispersers, employing various adaptations such as swimming, swarming, and sliding motility (35, 38–40). Additionally, it has been suggested that the ability of *Pseudomonas* to disperse even under dry conditions could be linked to their ability to produce biosurfactants, which can facilitate surface dispersal, especially under fluctuating hydration conditions (14, 41–43). However, the production of surfactants might be linked to specific habitats such as the rhizosphere (41, 44), and the presence of surfactants would therefore have to be proven under soil-like conditions.

In general, the dispersing bacterial communities of the soil and sediment compartments had a narrow phylogenetic distribution, and many dispersing taxa were shared between the compartments. These dispersers had several genera in common with dispersers from other plow layer soils (14, 15). Besides *Pseudomonas*, these were *Paenibacillus*, *Flavobacterium*, and *Janthinobacterium*, as well as *Rahnella* and *Pantoea*, the latter two belonging to the enterobacteria, a group identified as the most abundant disperser in a previous study (15). While *Pseudomonas* flagellar swimming dispersal and *Flavobacterium* gliding dispersal on surfaces have been studied extensively, mainly in pure cultures (18, 35, 45–48), there needs to be a greater focus on the mode of dispersal of other genera, e.g., *Pantoea*, which was found to be dominant in the deep matrix sediment at 300 to 350 cmbs in the present study.

Dispersal rates were severely inhibited under conditions dryer than −3.1 kPa, because a thinner liquid film on the ceramic surface prevents active dispersal, as previously demonstrated for both pure bacterial cultures (18, 35) and soil and lake microbial communities (14).

### Bacterial dispersers in preferential flow paths versus matrix sediments.

**FIG 7:**
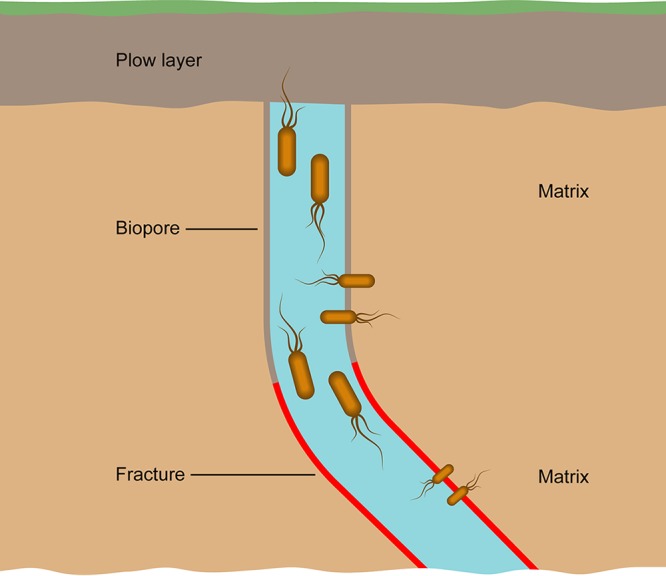
Conceptual model of preferential flow paths as facilitators of connectivity between communities and “hotspots” for the exchange of motile bacteria. The highest numbers of shared dispersers were observed along the preferential flow path (plow layer versus biopores and biopores versus fracture), fewer shared dispersers between the biopores and matrix, and almost none shared between the fracture and deep matrix. The size of the depicted bacteria represents the intensity of shared dispersers between compartments.

The high percentage of shared dispersers between the communities derived from the preferential flow path compartments compared to that from the matrix sediments indicated that the interchange of dispersing genera is more common along hydrologically connected compartments. This concept is illustrated in Fig. 7. As the preferential flow paths are enriched with dissolved nutrients, oxygen, and organic carbon transported from the surface by the flow of water (27, 49, 50), they can provide an attractive habitat for soil bacteria. While it has previously been shown that preferential flow paths have the potential to be a major route for the passive transport of bacteria (7, 16, 32, 33), the present results support the notion that part of the bacterial communities in the flow paths can also take advantage of active dispersal to spread through and colonize these habitats. This was especially interesting, as the benefit of active motility in the presence of water flow was not obvious. Indeed, it was expected that the benefit of active motility would be more prominent in the matrix soil where flow is absent.

The limited numbers of shared dispersers between the flow paths and the adjacent matrix sediments, especially at 300 to 350 cmbs (Table 1), may be due to limited connectivity. Given the small particle size of clay particles (<2 µm), the pore space in dense clay sediment is generally very small (7), probably impeding bacterial dispersal. Indeed, the porosity of the clay till at the present study site decreases with depth (51). Although some bacterial pure cultures are known to be able to swim through apertures as small as 1.1 µm (52), the small pore size and low connectivity of matrix clay tills likely form a barrier to the exchange of bacteria, in particular, at 300 to 350 cmbs. For comparison, deep fractures are reported to have apertures of 100 µm (22) and biopores may have diameters of 8 to 10 mm, leaving ample space for bacterial dispersal, subject to the presence of sufficient liquid films. Furthermore, fractures may be coated with metal oxide precipitates such as iron oxides, which can be almost impermeable to water (27, 53) and therefore probably also a hindrance for bacterial dispersal.

It is tempting to speculate that bacterial communities of dense clay matrixes exist as islands that have little contact with nearby communities. Water percolation through clay till matrix sediments is very limited (22, 23), thereby providing little input of nutrients and organic carbon to the bacteria. The small amount of nutrients in deep sediments (54) may also inhibit active dispersal, as nutrient limitation can greatly decrease the fraction of motile bacteria (55). We recognize that the method applied in this study is limited to the fraction of bacteria able to grow under the selected growth conditions, excluding the contribution of microbes that cannot be active under the conditions of our assay (e.g., strict anaerobes). Nonetheless, while the present study was limited to exploring subgroups of the total diversity of soil bacteria present along the preferential flow paths of a clay till depth profile, we believe that we are uncovering important processes relating dispersal potential and connectivity in the heterogeneous soil environment. The observed patterns of intensified cell exchange affected by dispersal potential and soil compartment should apply to other bacteria, and the principles of soil physics will apply irrespective of bacterial taxonomy.

### Dispersal at low matric potentials.

In unsaturated soil, low matric potentials are known to negatively affect bacterial dispersal (2, 18, 35). Here, the matric potential on the PSM was extended to −8.4 kPa, the lowest possible without using a pressurized version of the PSM (56), in order to investigate how low hydration conditions affect active dispersal of soil bacterial communities. The finding of active dispersal even at −8.4 kPa, albeit at a decreased rate, was surprising, because recent measurements of liquid films on the PSM have shown rapid thinning and disconnection of the liquid films at matric potentials exceeding −2.0 kPa (18), causing severe inhibition of dispersal, as demonstrated for bacterial pure cultures (18, 35, 46). However, due to residual surface roughness on the PSM, it is still possible to observe rare thicker liquid films (≥ 5 µm) at −2.0 kPa (18). Visual analysis of the agar plates suggested that dispersal at the lowest matric potentials of −6.3 to −8.4 kPa occurred along a few such narrow liquid film corridors on the rough surface. At a decreased matric potential (−4.1 to −8.4 kPa), *Bacillus* and *Pseudoduganella* rather than *Pseudomonas* were major dispersers in some replicates, indicating a stochastic element with regard to which bacteria disperse when water film thickness becomes limited. Due to the complex heterogeneous nature of soil, we speculate that there could also be some open dispersal corridors available in natural soil even under relatively dry conditions. One known option is the use of the thin liquid films surrounding fungal hyphae (i.e., fungal highways) (57, 58). It has been suggested that the abundance of mycelial networks in soil is part of the explanation for the maintenance of the otherwise costly flagella in soil bacteria (11, 59).

It has been claimed that active motility is limited in soil mainly due to dry and unsaturated conditions, which confines active dispersal to transient wet periods, e.g., during rain events (2, 7, 21). These claims have been supported in part by experimentation using the porous surface model, showing that bacterial flagellar motility is restricted to a narrow range of high matric water potentials (18, 35). While the relationship between matric potential and liquid film thickness on the PSM differs from that in soil, it is relevant to ascertain if the range of matric potentials found in soil is compatible with flagellum-powered swimming. According to data from the Danish Pesticide Leaching Assessment Programme (PLAP) (60, 61), in fractured clay till, which is a common soil type in Denmark (26), the matric potential of Danish agricultural top soils can fluctuate between −5 and −1,500 kPa, while deeper clayey matrix sediments (from 60 cmbs and down) remain water saturated (∼0 kPa) most of the time (61, 62; D. Nagy, A. E. Rosenbom, B. V. Iversen, M. Jabloun, F. Plauborg, manuscript in preparation). There should therefore be sufficient liquid films in subsurface clay till to allow active dispersal unless low pore connectivity and fracture coatings create physical barriers that cannot be overcome.

### Conclusions.

This study demonstrated that different compartments of a heterogeneous clay till depth profile harbor bacterial communities that are capable of dispersing under low hydration conditions. The dispersers show narrow phylogenetic diversity and are dominated by pseudomonads and enterobacteria. Active dispersal occurred even within thin and poorly connected liquid films on the surface of the PSM at matric potentials of −6.3 to −8.4 kPa. These results indicate that active dispersal ability is widespread in soil and sediment communities. An increased proportion of disperser ASVs shared between highly connected compartments (e.g., preferential flow paths) points to a role for active dispersal in the spread through, and colonization of, these habitats. Fewer shared disperser ASVs between the preferential flow paths and the matrix sediments illustrated that low porosity of clay tills and metal oxide-coated fracture walls might be barriers to the exchange of bacteria, leaving matrix bacterial communities relatively isolated.

## MATERIALS AND METHODS

### Soil sampling.

Soils were sampled over a 3-day period in September 2016 from an agricultural field (Anthric Luvisol) in Lund, Denmark (55°14′49′′N, 12°17′24′′E) (51). The adjacent field was recently included in the Danish Pesticide Leaching Assessment Programme (PLAP; http://pesticidvarsling.dk) (51). The soil is characterized by clay till and boulder clay, with a very pronounced fracture system down to at least a 6-m depth. While the biopores, dominating the top 150 cm below the surface (cmbs), mainly consist of earthworm burrows and decayed root channels, the fractures below are mainly of tectonic origin.

A multibench excavation down to a 6-m depth allowed the sampling of sediment from different depths. Soil was sampled from the plow layer (0 to 20 cmbs), biopores (80 to 120 cmbs), matrix sediment next to the biopores (80 to 120 cmbs), oxidized iron-rich red fractures (300 to 350 cmbs), and matrix sediment next to these fractures (300 to 350 cmbs) (Fig. 1). Soils were collected as composite samples, i.e., as small subsamples combined into one pooled sample for each of the five soil and sediment compartments. One composite sample equaled ca. 15 to 30 subsamples per soil compartment, except for the biopore samples, which consisted of ca. 70 subsamples. The subsamples were combined into one composite sample per compartment to ensure sufficient soil from biopores and fractures for further analysis. Samples were secured by carefully removing the outer layer of the soil profile with a knife to avoid cross contamination. Then, the freshly exposed soil and sediment were subsampled (carefully scraped off) with a small spoon and stored at 5°C.

### Extraction of soil bacteria.

The soil and sediment samples from each compartment were homogenized by sieving (2 mm), and mass reduction for laboratory subsampling was performed by bed blending, as described in reference 63 and by Kardanpour et al. (64). This resulted in a 25-g composite soil or sediment sample for each experimental setup.

The soil bacterial community from each compartment was extracted using Nycodenz density gradient centrifugation as described in reference 65, except for the final cell density determination, which was performed directly using a Thoma counting chamber. Cell densities of the extracts were adjusted to 0.8 × 10^6^ cells · µl^−1^ in 0.9% NaCl solution. The soil bacterial extracts were kept at 4°C overnight before inoculation on the ceramic discs of the extended porous surface model system.

### Dispersal potential of environmental communities using the extended porous surface model.

An extended version of the porous surface model (PSM) (14) was used in which the original PSM model (46) was further developed to encompass the dispersal of nonfluorescent complex communities extracted from environmental samples. The method allows communities to disperse under controlled hydration conditions from the center of a porous ceramic disc (diameter, 41.3 mm; thickness, 7.1 mm; maximum pore size, <1.5 µm; 100-kPa bubbling pressure; Soilmoisture Equipment Corp., Santa Barbara, CA), mimicking a rough soil surface. Imposing suction on the ceramic disc allows for precise control of the liquid film thickness on its surface. The liquid medium used in the PSM was 25% R2B (Alpha Biosciences, Baltimore, MD). Each experimental setup allowed for the parallel incubation of 9 to 11 PSMs.

Each PSM was inoculated with 10 µl of bacterial extract placed as 1-µl drops at the center of the ceramic disc. Although the inocula for the PSM were adjusted to the same cell densities according to Thoma counts, the cultivable fraction was generally lower in deep sediment samples than in plow layer and biopore soil. CFU numbers were highest in the plow layer (ca. 11,250 CFU) and biopores (130,000 CFU), and decreased in the matrix at 80 to 120 cmbs (1,000 CFU), red fractures (1,125 CFU), and matrix sediment from 300 to 350 cmbs (750 CFU). Colonies were enumerated on 25% R2A plates (Fluka R2A; Sigma-Aldrich, St. Louis, MO) after incubation at 25°C for 48 to 72 h. All plates were amended with 100 mg · liter^−1^ natamycin to inhibit fungal growth (Delvocid; DSM Food Specialties, Delft, The Netherlands).

After inoculation, the discs were brought to a matric potential of −0.5 or −3.1 kPa and incubated at room temperature for 24 or 48 h before sampling. After incubation, the bacteria were recovered from the surface of the ceramic disc by means of an agar plate lift. This is described in detail by Krüger et al. (14). In brief, to visualize the colonization on the ceramic disc, a series of agar plates were used to cover different sections of the ceramic surface. The agar plate series consisted of small flat 25% R2A plates containing 20 g agar · liter^−1^ (Star Dish; diameter, 40 mm; height, 12.5 mm; Phoenix Biomedical Products, Mississauga, Canada), with holes in four sizes. Sampling was achieved by starting with the plate with the largest hole size, 25 mm, followed by 20, 15, and 11.5 mm, and ending with the pressing of a full agar plate (full plate). The extent of colonization of the ceramic disc was quantified by evaluating the coverage of bacterial growth on the individual agar plates after 72 h of incubation at 25°C and dividing it into four categories: 1% to 25%, 26% to 50%, 51% to 75%, and 76% to 100% coverage.

For each series of five pressed plates, the fastest-dispersing bacteria from the environmental communities, i.e., the colonies of the pressed agar plate furthest from the point of inoculation (the plate with the largest hole size) that presented growth (referred to as the “dispersers” or “dispersing community”), and the total community present on the full agar plates were identified by 16S rRNA gene amplicon sequencing. The full plate represented the cultivable community developing on an agar plate covering the entire ceramic plate, i.e., both dispersing and nondispersing bacteria. Additionally, for each separate experiment and soil, a no-motility reference plate, or “reference plate,” was made by drop-plating 10 µl of each inoculum directly onto the center of a small 25% R2A plate with 20 g agar · liter^−1^, which provided conditions that are not conductive for flagellar motility and are not influenced by the PSM (34). All bacteria were washed off the agar plates with 0.9% NaCl according to the procedure described by Krüger et al. (14). For comparisons with the dispersed communities, total communities present on the full plates were generally preferred, as they captured the double cultivation step (both on the PSM and on the agar plates). However, the reference plates were also valuable because they gave an indication of what could be cultivated upon direct inoculation on the agar plates. The cell suspensions from the pressed plates and the reference plates (plate wash), as well as the original Nycodenz extracts, were stored at −80°C before further processing.

### Porous surface model with increasingly negative matric potentials.

To achieve matric potentials down to −8.4 kPa, the PSM assembly was slightly modified. To limit the amount of air entering the system, the PSM tubing was tightened and partly replaced with stainless steel. To further limit the formation of air bubbles that can form in the medium at lowered matric potentials, the ceramic plates were degassed for 24 h using a vacuum pump, and the 25% R2B medium was degassed for 20 min in an ultrasound bath. PSMs were assembled submerged in degassed medium.

### DNA extraction and sequencing.

DNA was extracted using the DNeasy PowerLyzer PowerSoil kit (Qiagen; Hilden, Germany) according to the manufacturer’s protocol with a few adjustments, as in Krüger et al. (14). The DNA concentrations were measured on Qubit 2.0 (Life Technologies, Invitrogen; Carlsbad, CA), and samples were stored at −80°C until sequencing. The DNA was PCR amplified using the primer set 341F (5′-CCTACGGGNGGCWGCAG-3′) and 806R (5′-GACTACHVGGGTATCTAATCC-3′) (66) targeting the hypervariable V3-V4 regions of bacterial 16S rRNA genes. The purified PCR products (2 × 300-bp reads) were sequenced on the Illumina MiSeq platform by Macrogen (Seoul, South Korea).

The raw 16S rRNA gene amplicon sequences were processed using the DADA2 pipeline (67) with default parameters. Sequence classification was based on the SILVA prokaryotic reference database, version 123 (68). A total of 7.2 million sequences passed the filtering steps, representing an average of 60,500 sequences per sample.

### Data analysis and statistical methods.

Data analysis of sequences and statistics were performed in R (69). The Shannon diversity index was calculated using the plot_richness function in the phyloseq package (70). Faith’s phylogenetic diversity (PD) was calculated with the pd.query function in the PhyloMeasures package (71). Prior to calculating the PD, samples were rarefied to an even depth (mean of 10 iterations) using the rarefy_even_depth function in the phyloseq package. Heatmaps were plotted using the amp_heatmap function in the ampvis2 package (72). Venn diagrams were plotted using the function venn from the gplots package (73). Nonmetric multidimensional scaling (NMDS) ordination was undertaken on Bray-Curtis dissimilarities using the ordinate function in the phyloseq package. PERMANOVA and analysis of multivariate homogeneity of group dispersions (variances) were computed using the adonis and betadisper functions in the vegan (2.4-6) package (74), with 999 permutations. Differences in the proportions of shared ASVs between communities were tested using Fisher’s exact tests in R (69).

Additional statistical analysis was undertaken using SigmaPlot 13 (Systat Software, Inc., San Jose, CA).

Differences in Shannon diversity indices between total communities and the fastest dispersers were tested using one-tailed one-sample *t* tests (testing for subtracted differences greater than zero). The effects of matric potentials were tested using two-tailed *t* tests. *P* values of <0.05 were considered significant.

### Accession number(s).

All sequencing data have been deposited as an NCBI BioProject under accession number PRJNA483533.

## Supplementary Material

Supplemental file 1
