# Supplementary material for: Bacterial Dispersers along Preferential Flow Paths of a Clay Till Depth Profile
Source: Appl Environ Microbiol. 2019 Mar 6;85(6):e02658-18. doi: 10.1128/AEM.02658-18 (PMC6414393; doi:10.1128/AEM.02658-18)
Supplement: Supplemental file 1 [file AEM.02658-18-s0001.pdf]

Supporting Information:

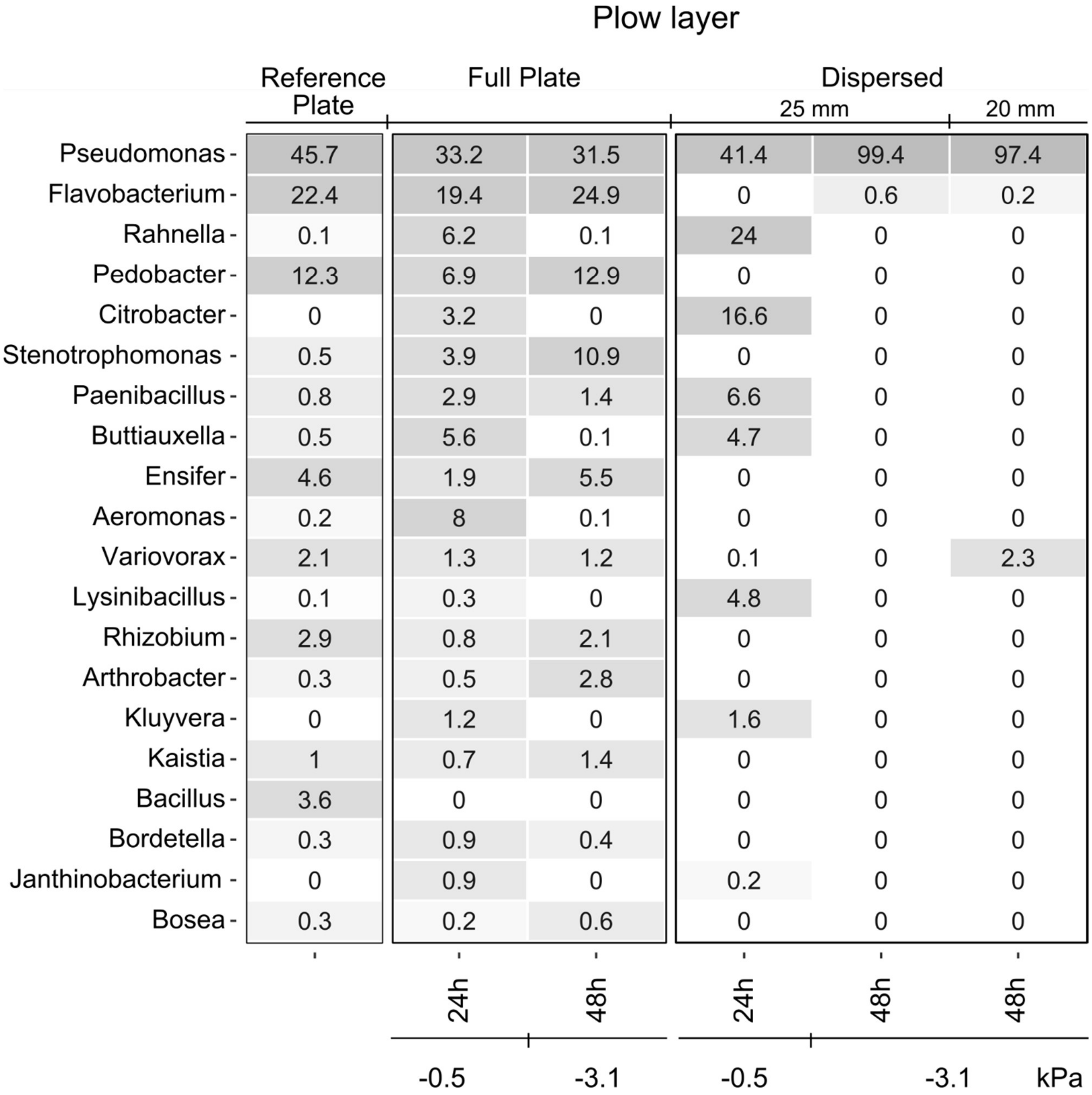

Figure S1. Heatmap of the relative abundances of the 20 most dominant genera across communities derived from a soil extract from plow layer soil, and differing in their dispersal after being incubated at different prescribed matric potentials for 24 h or 48 h. Columns present the average results for triplicate communities except for the restricted-motility control (reference plate; *n*=2), and the fastest-dispersed community at -3.1 kPa at 48 h recovered from the 20 mm (n=2), and 25 mm (n=1) sections.

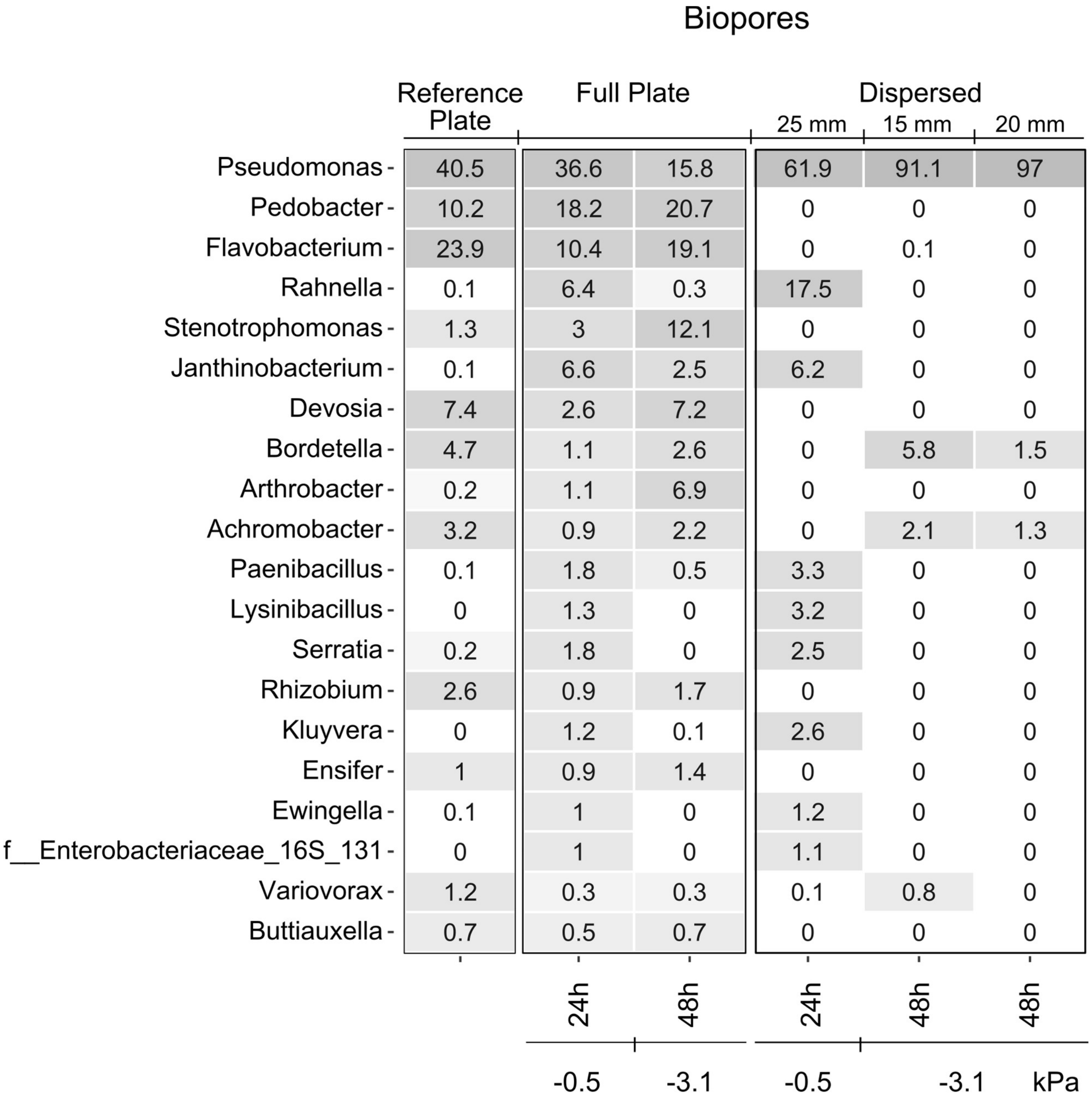

Figure S2. Heatmap of the relative abundances of the 20 most dominant genera across communities derived from a soil extract from biopores, and differing in their dispersal after being incubated at different prescribed matric potentials for 24 h or 48 h. Columns present the average results for triplicate communities except for the restricted-motility control (reference plate; *n*=1), the total community on the full plate at -3.1 kPa at 48 h (n=4), and the fastest-dispersed community at -3.1 kPa at 48 h, recovered from the 15 mm (n=2) and 20 mm (n=2) sections.

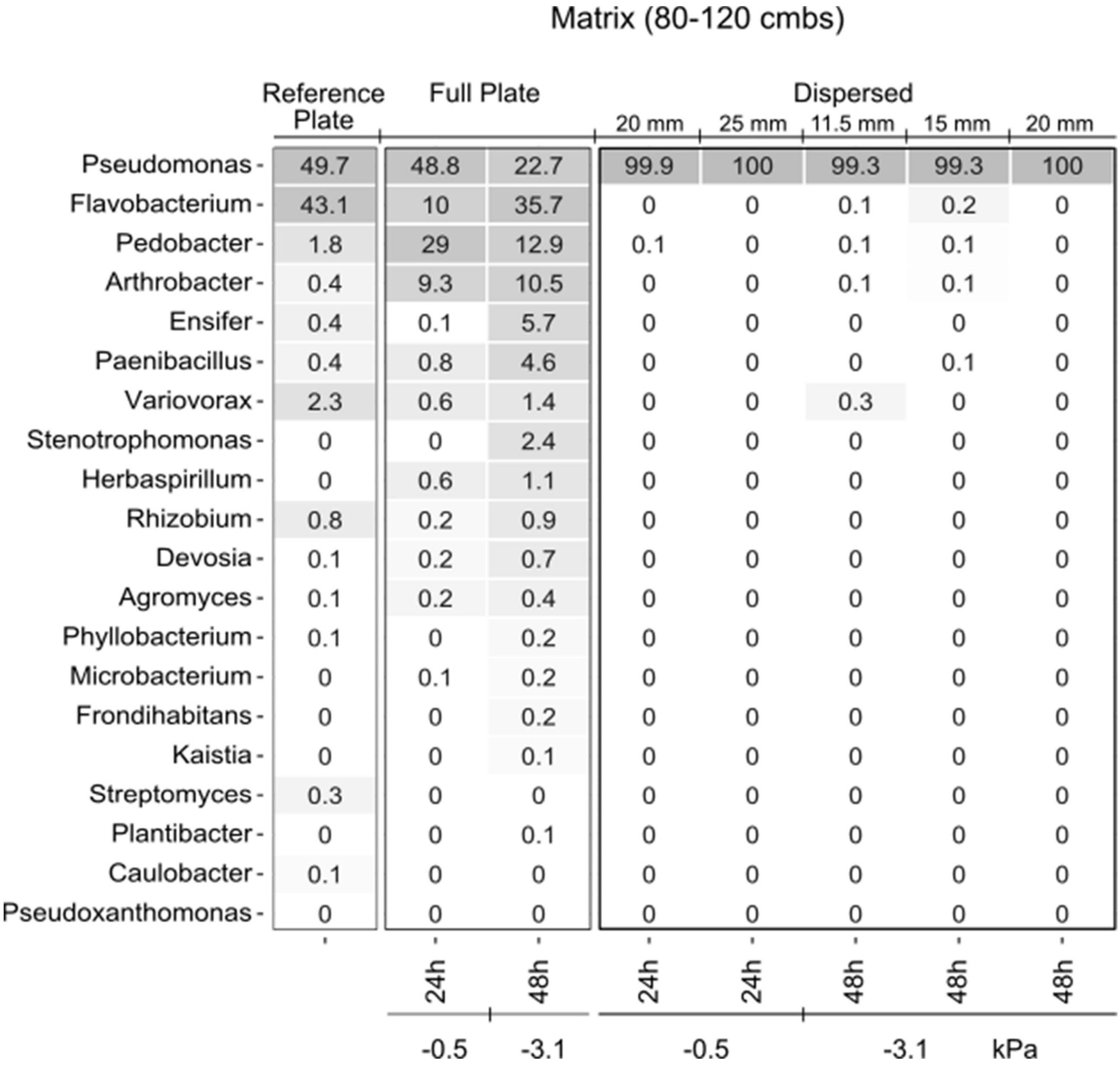

Figure S3. Heatmap of the relative abundances of the 20 most dominant genera across communities derived from a soil extract from matrix sediments (80-120 cmbs), and differing in their dispersal after being incubated at different prescribed matric potentials for 24 h or 48 h. Columns present the average results for triplicate communities except for the restricted-motility control (reference plate; *n*=1), the total community on the full plate at -3.1 kPa at 48 h (n=4), the fastest-dispersed community at -0.5 kPa at 24 h recovered from the the 20 mm (n=2) and 25 mm

(n=1) sections, and -3.1 kPa at 48 h, recovered from the 11.5 mm (n=2), 15 mm (n=1) and 20 mm (n=1) sections

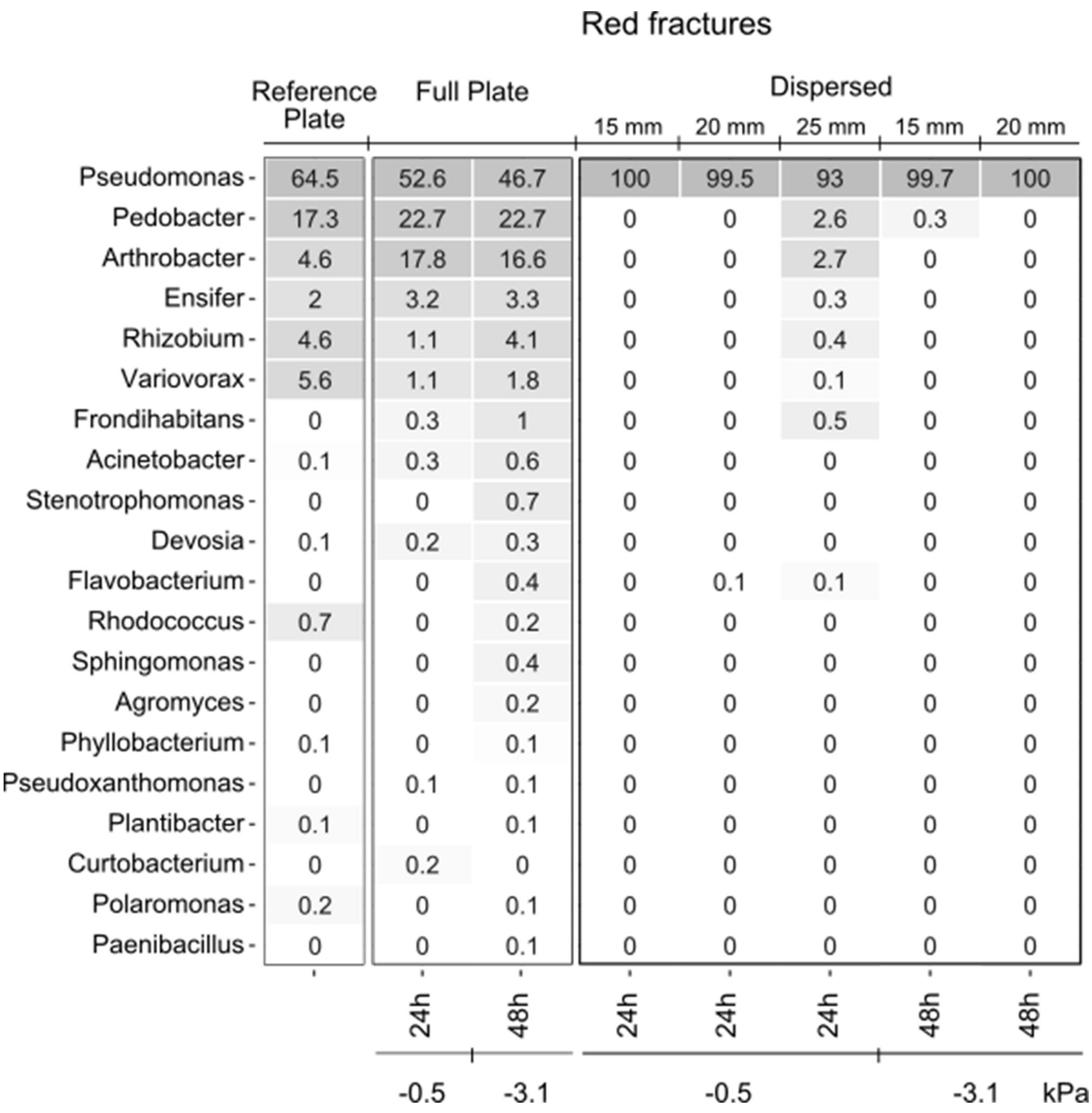

Figure S4. Heatmap of the relative abundances of the 20 most dominant genera across communities derived from a soil extract from red fractures (300-350 cmbs), and differing in their dispersal after being incubated at different prescribed matric potentials for 24 h or 48 h. Columns present the average results for triplicate communities except for the restricted-motility control

(reference plate; *n*=1), the total community on the full plate at -3.1 kPa at 48 h (n=4), the fastest-dispersed community at -0.5 kPa at 24 h recovered from the 15 mm (n=1), 20 mm (n=1) and 25 mm (n=1) sections, and -3.1 kPa at 48 h, recovered from the 15 mm (n=3) and 20 mm (n=1) sections

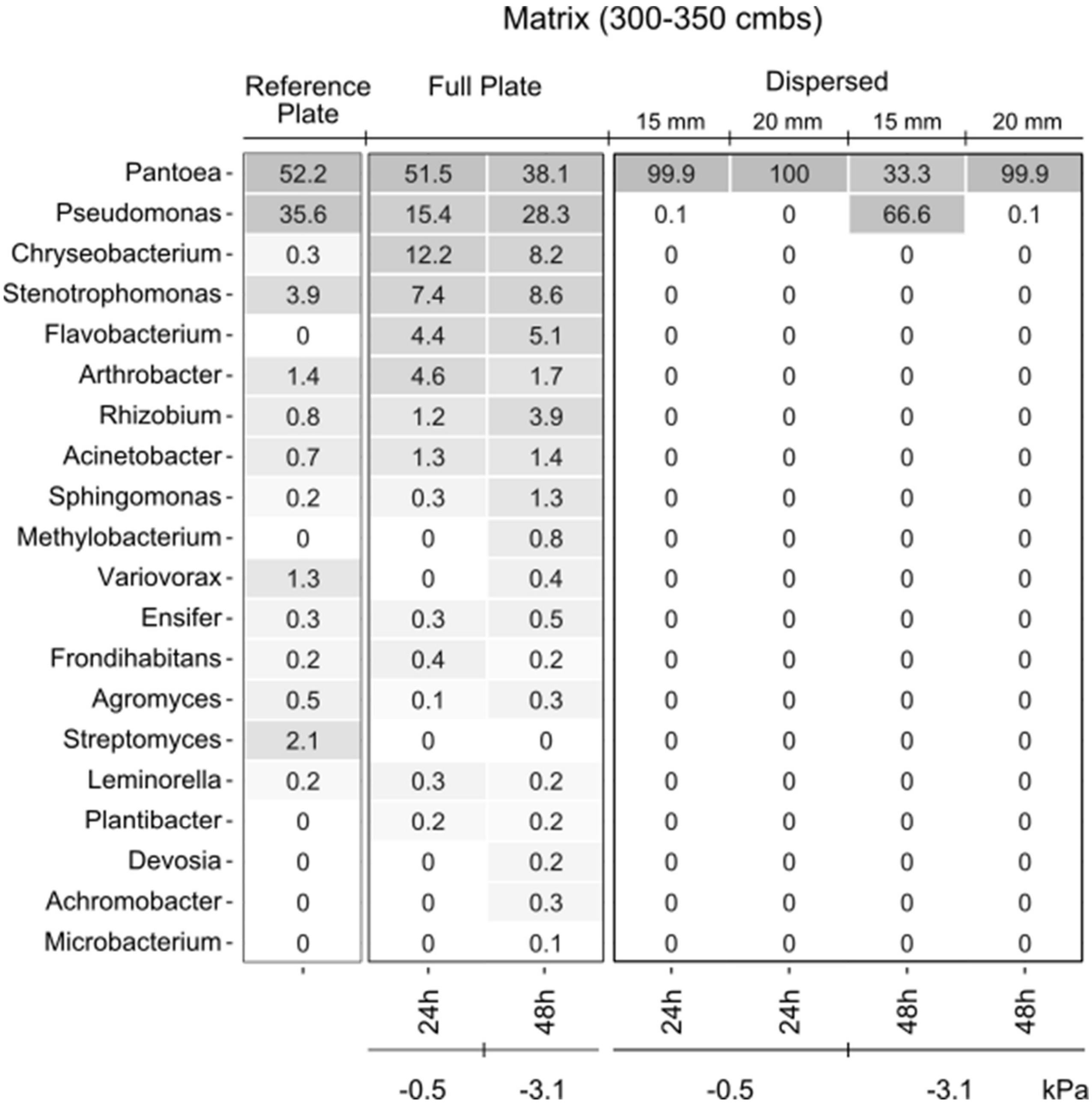

Figure S5. Heatmap of the relative abundances of the 20 most dominant genera across communities derived from a soil extract from matrix sediment (300-350 cmbs), and differing in their dispersal after being incubated at different prescribed matric potentials for 24 h or 48 h. Columns present the average results for triplicate communities except for the restricted-motility control (reference plate; *n*=1), the total community on the full plate at -3.1 kPa at 48 h (n=4), the

fastest-dispersed community at -0.5 kPa at 24 h recovered from the 15 mm (n=1) and 20 mm (n=2) sections, and -3.1 kPa at 48 h, recovered from the 15 mm (n=3) and 20 mm (n=1) sections

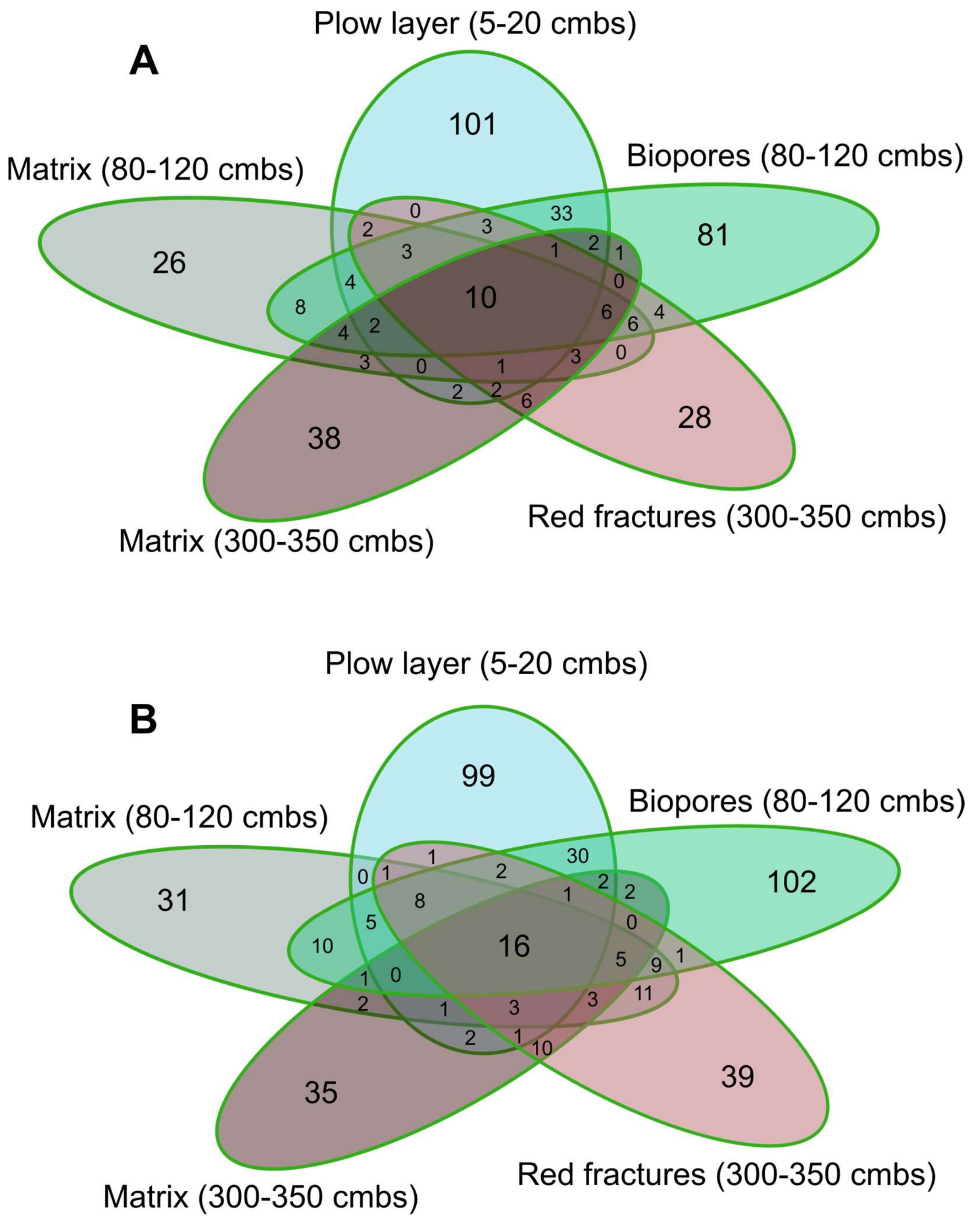

Figure S6. Venn diagrams depicting the shared and separate unique ASVs between five bacterial communities (full plate) from five compartments of a well-defined soil profile. A) shows the five communities exposed to -0.5 kPa for 24 h and contains a total of 381 unique ASVs. B) shows the five communities exposed to -3.1 kPa for 48 h and contains a total of 434 unique ASVs.

Plow layer	Soil	Nycodenz	Reference Plate	Full Plate 24h (-0.5 kPa)	Full Plate 48h (-3.1 kPa)
Pseudomonas	0.4	3.2	39.3	33.2	31.5
Flavobacterium	1.4	1.5	26.3	19.4	24.9
Pedobacter	0.1	0.7	16.7	6.9	12.9
Stenotrophomonas	0	0	0.5	3.9	10.9
Ensifer	0	0.7	3.9	1.9	5.5
Aeromonas	0	0	0.1	8	0.1
Rahnella	0	0	0.2	6.2	0.1
Buttiauxella	0	0	1	5.6	0.1
Lysobacter	3.9	4.2	0	0	0
Paenibacillus	0	0.5	0.2	2.9	1.4
Sphingomonas	1.7	8	0	0	0
Arthrobacter	0.6	0.7	0.3	0.5	2.8
Rhizobium	0	0.4	3.1	0.8	2.1
Variovorax	0.1	0.6	2.7	1.3	1.2
Citrobacter	0	0	0	3.2	0
Kaistia	0	0	1.2	0.7	1.4
Blastocatella	2.1	0.5	0	0	0
Arenimonas	1.6	1.7	0	0	0
Gaiella	1.1	2.6	0	0	0
Nitrospira	1.5	0.1	0	0	0

Figure S7. Heatmap of the relative abundances of the 20 most dominant genera across communities derived from a soil extract from plow layer soil. Full plate communities were recovered on plates after incubation at prescribed matric potential for 24 h or 48 h. Columns present the average of triplicate communities, except for the Nycodenz extraction (n=2) and the motility restricted control (Reference Plate; n=2).

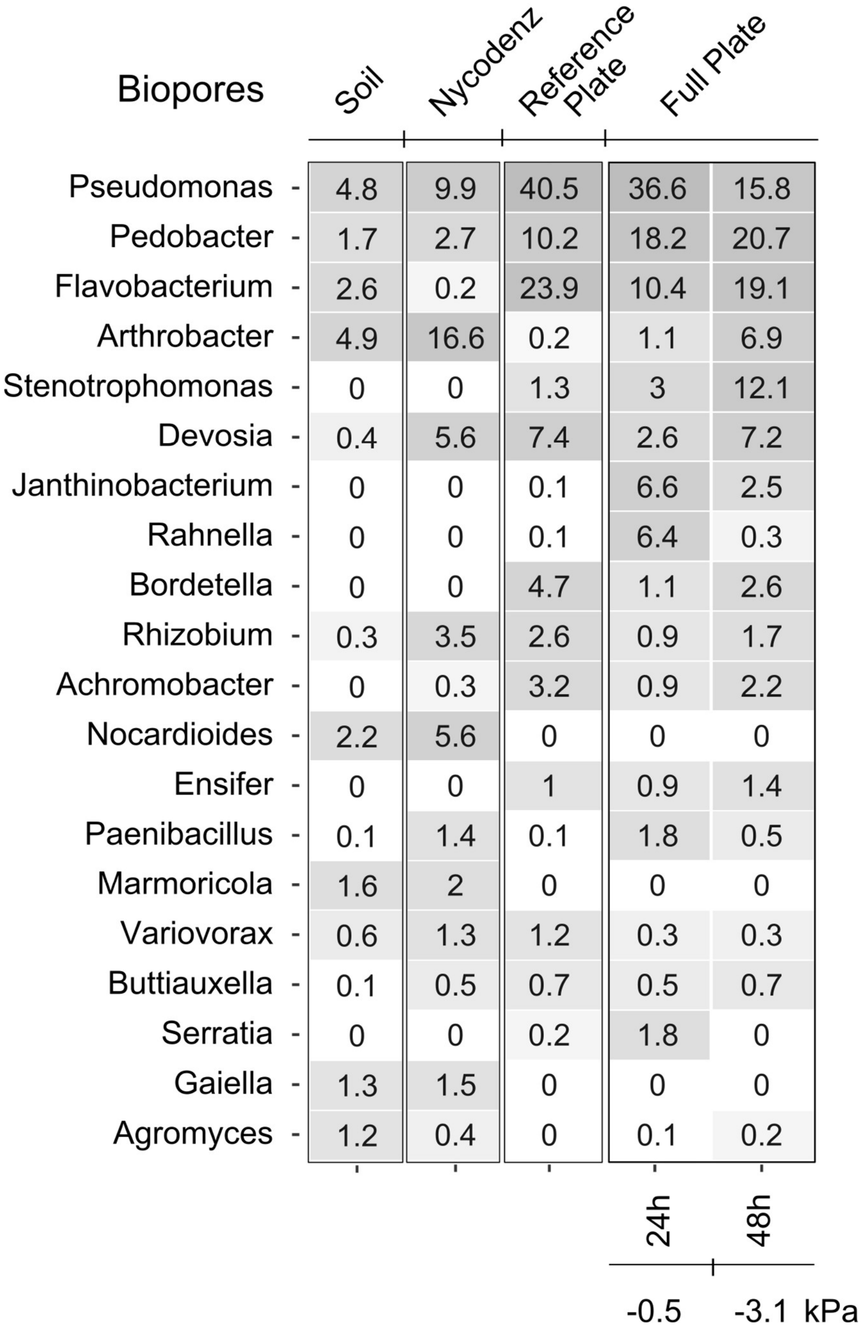

Figure S8. Heatmap of the relative abundances of the 20 most dominant genera across communities derived from a soil extract from biopore soil linings. Full plate communities were recovered on plates after incubation at prescribed matric potential for 24 h or 48 h. Columns present the average of triplicate communities, except for the Nycodenz extraction (n=1) and the motility restricted control (Reference Plate; n=1).

Table S1. Total amplicon sequence variants of cultured communities (full plate, reference plate and fastest dispersers) present in the five soil and sediment compartments. ASVs are totals for -0.5 kPa 24 h and -3.1 kPa 48 h		
	**Unique ASVs identified at genus level**	**Unidentified ASVs**
Plow layer	68	59
Biopores	73	58
Matrix (80-120)	46	10
Red fractures	45	22
Matrix (300-350)	38	18
All samples, all compartments	109	161

Table S2. Comparison between identified genera in cultured communities (full plates) and the original soil communities			
	**Total genera present**	**Number of genera also present in the original soil community at abundance >0.1 %**	**Percentage of genera in original soil community with abundance >0.1 %, recovered on full plates**
Biopores -0.5 kPa 24 h (Full plate)	47	13	23.6 %
Biopores -3.1 kPa 48 h (Full plate)	50	15	27.7 %
Biopore soil (abundance >0.1%)	55		
Plow layer -0.5 kPa 24 h	45	7	15.2 %
Plow layer -3.1 kPa 48 h	45	4	8.7 %
Plow layer soil (abundance >0.1 %)	46		

Table S3. Comparison between ASVs in cultured communities (full plates) and the original soil communities		
	**Total ASVs present on full plates**	**Percentage of original soil community %**
Biopores -0.5 kPa 24 h and -3.1 kPa 48 h	258	10 %
Plow layer -0.5 kPa 24 h and -3.1 kPa 48 h	260	1 %

**Table S4. Shared amplicon sequence variants between the fastest dispersers in the five soil and sediment compartments. -0.5 kPa 24 h**	
**Combinations**	**Shared ASVs**
**Shared between preferential flowpath samples**	
Plow layer: biopores	28
Biopores: red fractures	27
Plow layer: red fractures	14
Plow layer: biopores: red fractures	12
**Flowpath *vs.* matrix**	
Biopores: matrix (80-120)	16
Red fractures: matrix (300-350)	3

**Table S5. Shared amplicon sequence variants between the fastest dispersers in the five soil and sediment compartments. -3.1 kPa 48 h**	
**Combinations**	**Shared ASVs**
**Shared between preferential flowpath samples**	
Plow layer: biopores	15
Biopores: red fractures	11
Plow layer: red fractures	15
Plow layer: biopores: red fractures	11
**Flowpath *vs.* matrix**	
Biopores: matrix (80-120)	18
Red fractures: matrix (300-350)	3

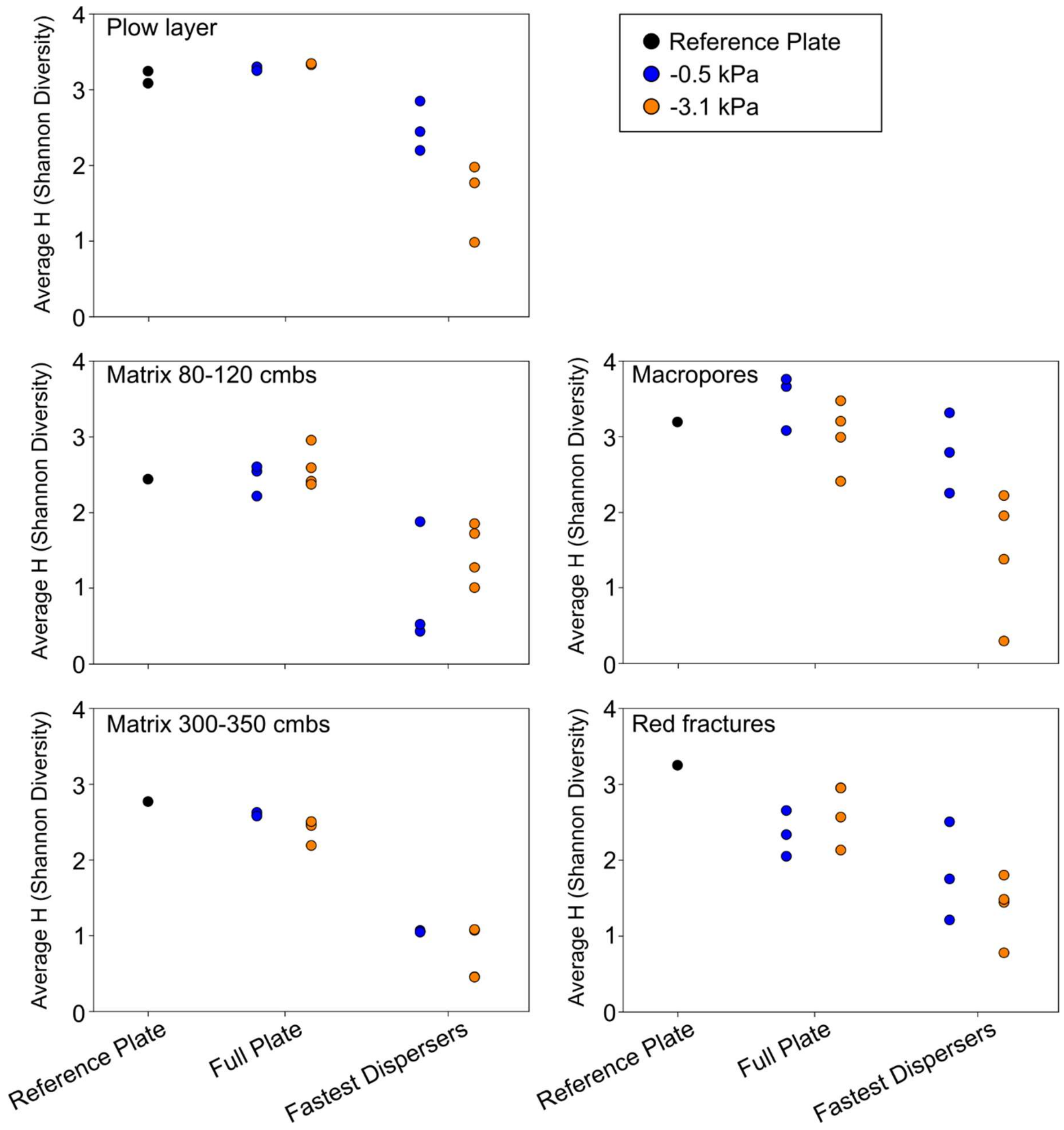

Figure S9. Estimates of alpha diversity (Shannon diversity index) for communities derived from five compartments of a well-defined soil profile after 24 h (-0.5 kPa) or 48 h (-3.1 kPa) of incubation at different prescribed matric potentials. For each matric potential, the results for the total community recovered from the full agar plate (full plate) and the fastest-dispersed community are presented. A restricted-motility control (reference plate) is also included. Replicates are depicted as separate dots, n=3, except for -3.1 kPa for matrix 80-120, matrix 300-350, biopores and red

fractures where n=4. The values depicted are not rarified, but rarifying 10x provided similar results.

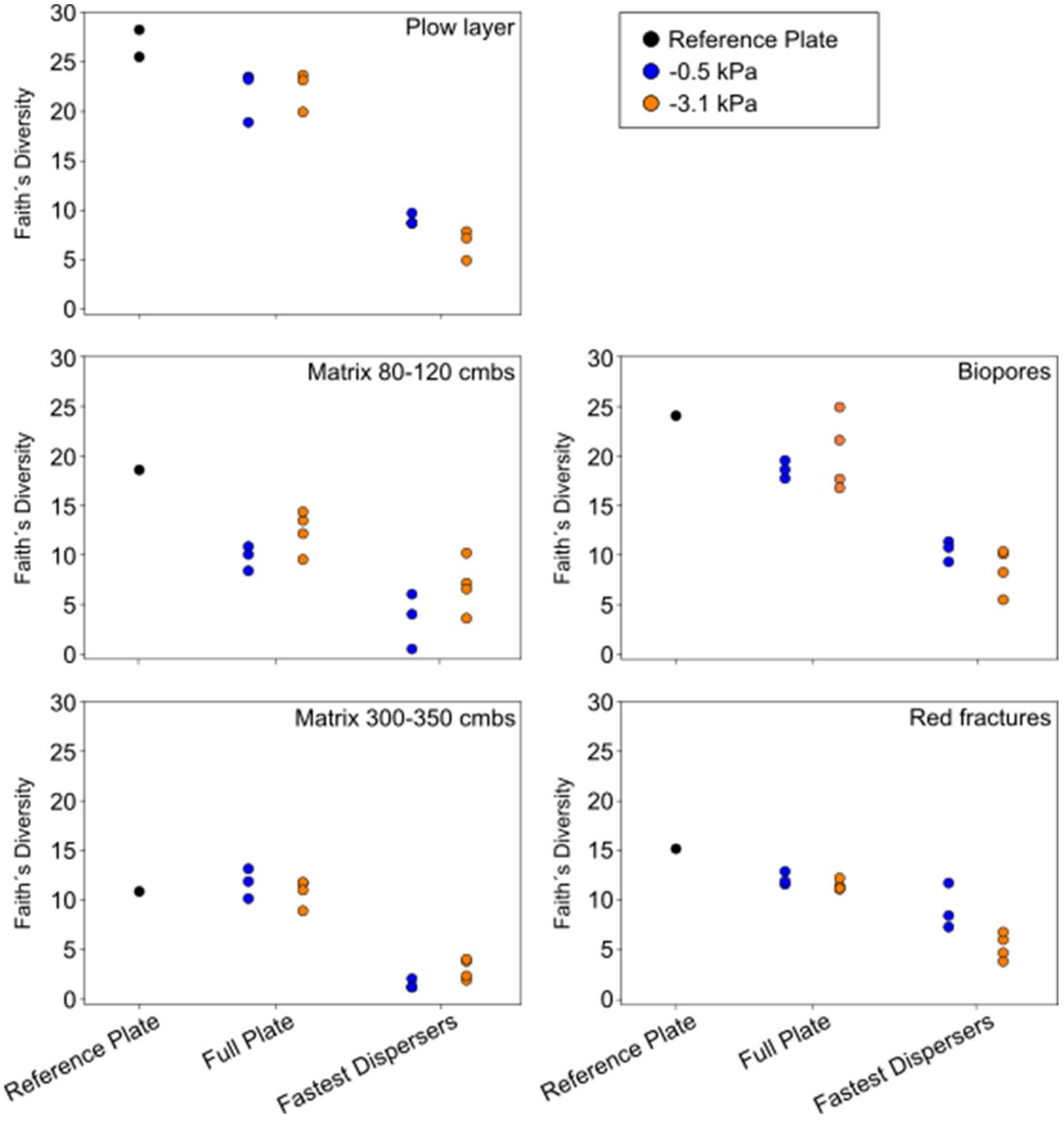

Figure S10. Estimates of alpha diversity (Faith's phylogenetic diversity index) for communities derived from five compartments of a well-defined soil profile after 24 h (-0.5 kPa) or 48 h (-3.1 kPa) of incubation at different prescribed matric potentials. For each matric potential, the results for the total community recovered from the full agar plate (full plate) and the fastest-dispersed community are presented. A restricted-motility control (reference plate) is also included.

Replicates are depicted as separate dots, n=3, except for – 3.1 kPa for matrix 80-120, matrix 300-350, biopores and red fractures where n=4. The reported values are averages obtained for 10 random rarefactions.

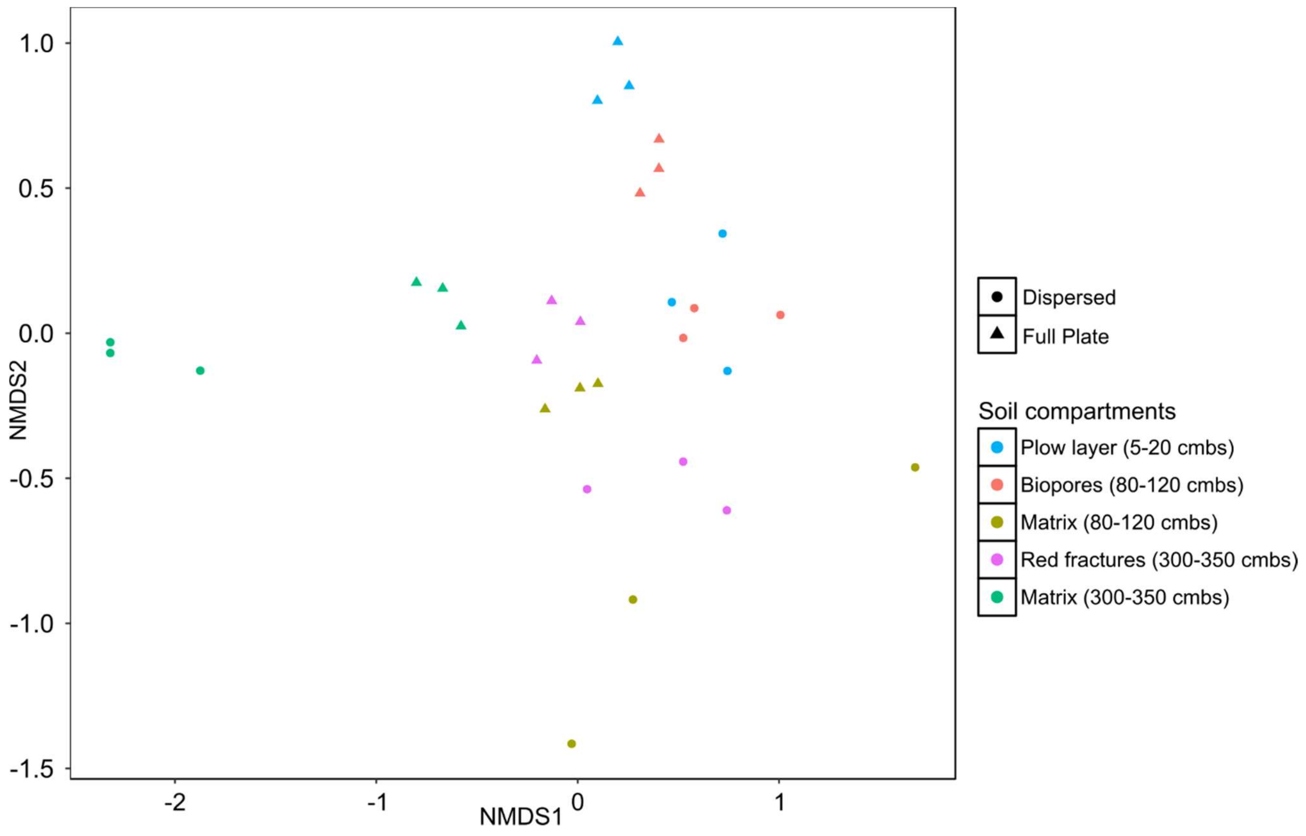

Figure S11. NMDS plot of the composition of the total communities (triangles) and dispersed communities (circles) derived from five compartments of a well-defined soil profile.

Stress = 0.1491. Bray-Curtis dissimilarities calculated from 16S rRNA genes. The communities were recovered from the PSM experiments on the full agar plates (full plate) and the community the furthest from the center (dispersed). The communities were exposed to matric potential -0.5 kPa for 24 h. Replicates are depicted as separate dots.

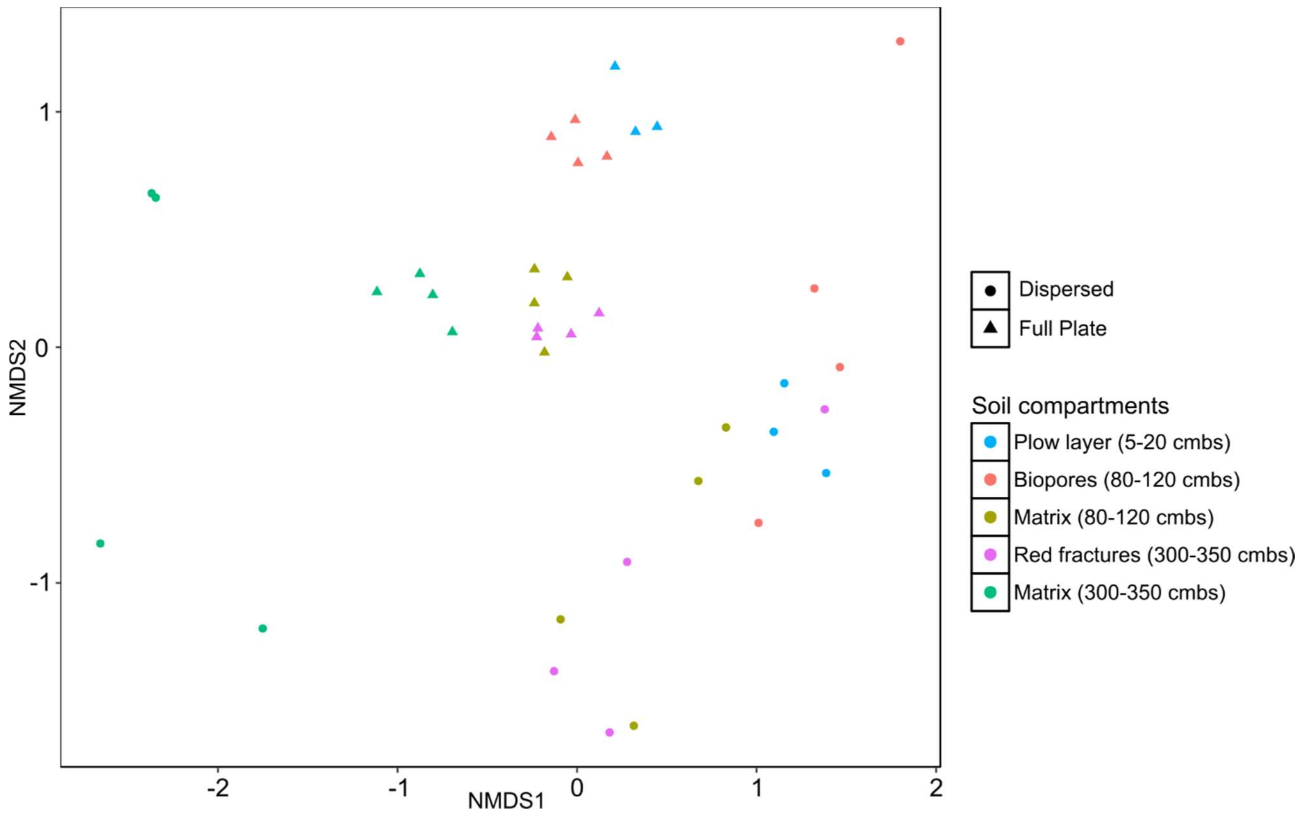

Figure S12. NMDS plot of the composition of the total communities (triangles) and dispersed communities (circles) derived from five compartments of a well-defined soil profile.

Stress = 0.1491. Bray-Curtis dissimilarities calculated from 16S rRNA genes. The communities were recovered from the PSM experiments, on the full agar plates (full plate) and the community the furthest from the center (dispersed). The communities were exposed to matric potential -3.1 kPa for 48 h. Replicates are depicted as separate dots.

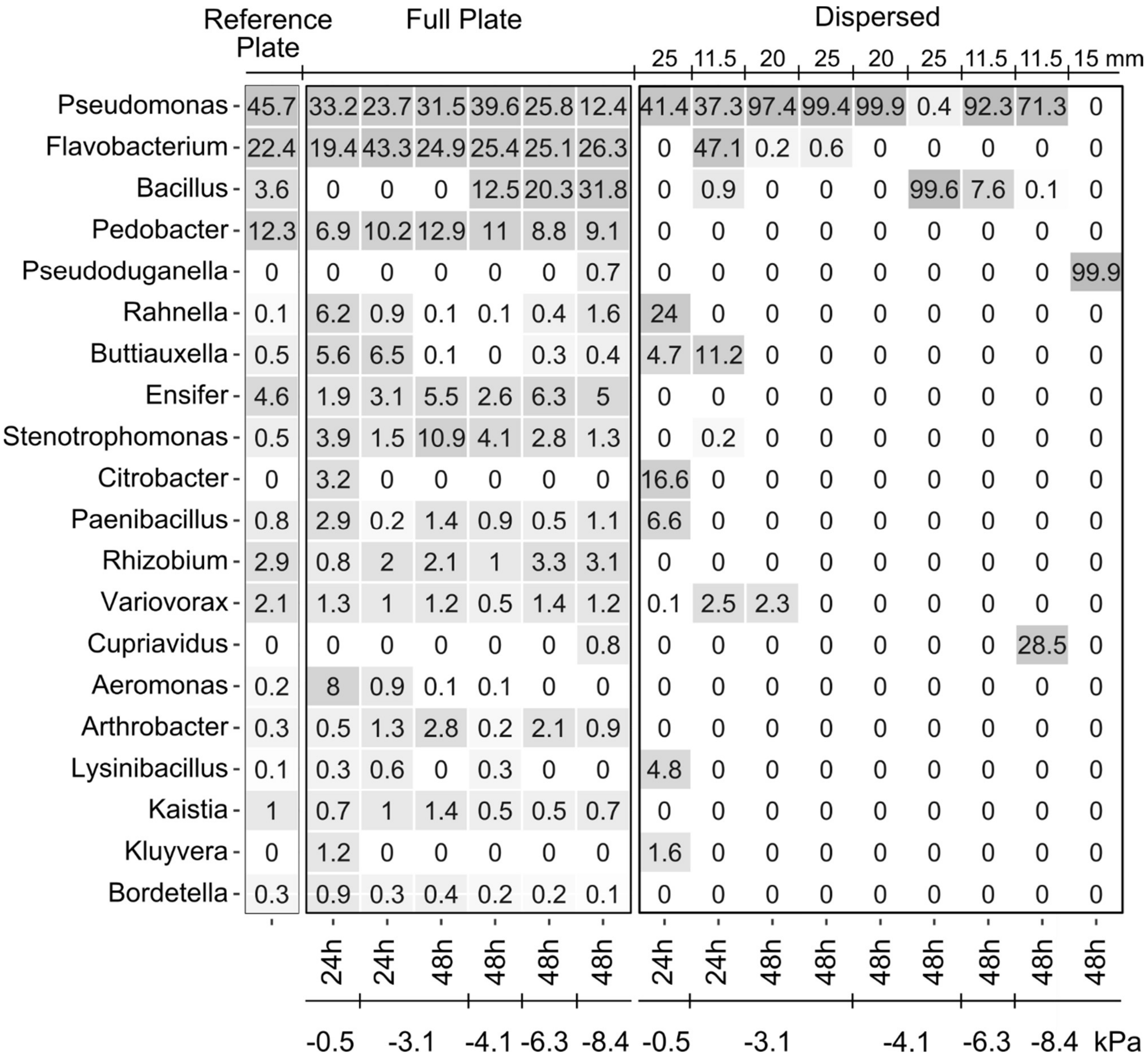

Figure S13. Heatmap of the relative abundances of the 20 most dominant genera across communities derived from a soil extract from plow layer soil, and differing in their dispersal after being incubated at different prescribed matric potentials for 24 h or 48 h. Columns present the average results for duplicate communities, except for the total community on the full plate at -0.5 kPa at 24 h (n=3), -3.1 kPa at 24 h (n=3), 48 h (n=3), the fastest-dispersed community at -4.1 kPa at 48 h recovered from the 20 mm (n=1) and 25 mm (n=1) sections, at -6.3 kPa at 48 h, recovered

from the 11.5 mm (n=1) section, and at -8.4 kPa at 48 h recovered from the 11.5 mm (n=1) and 15 mm (n=1) sections.

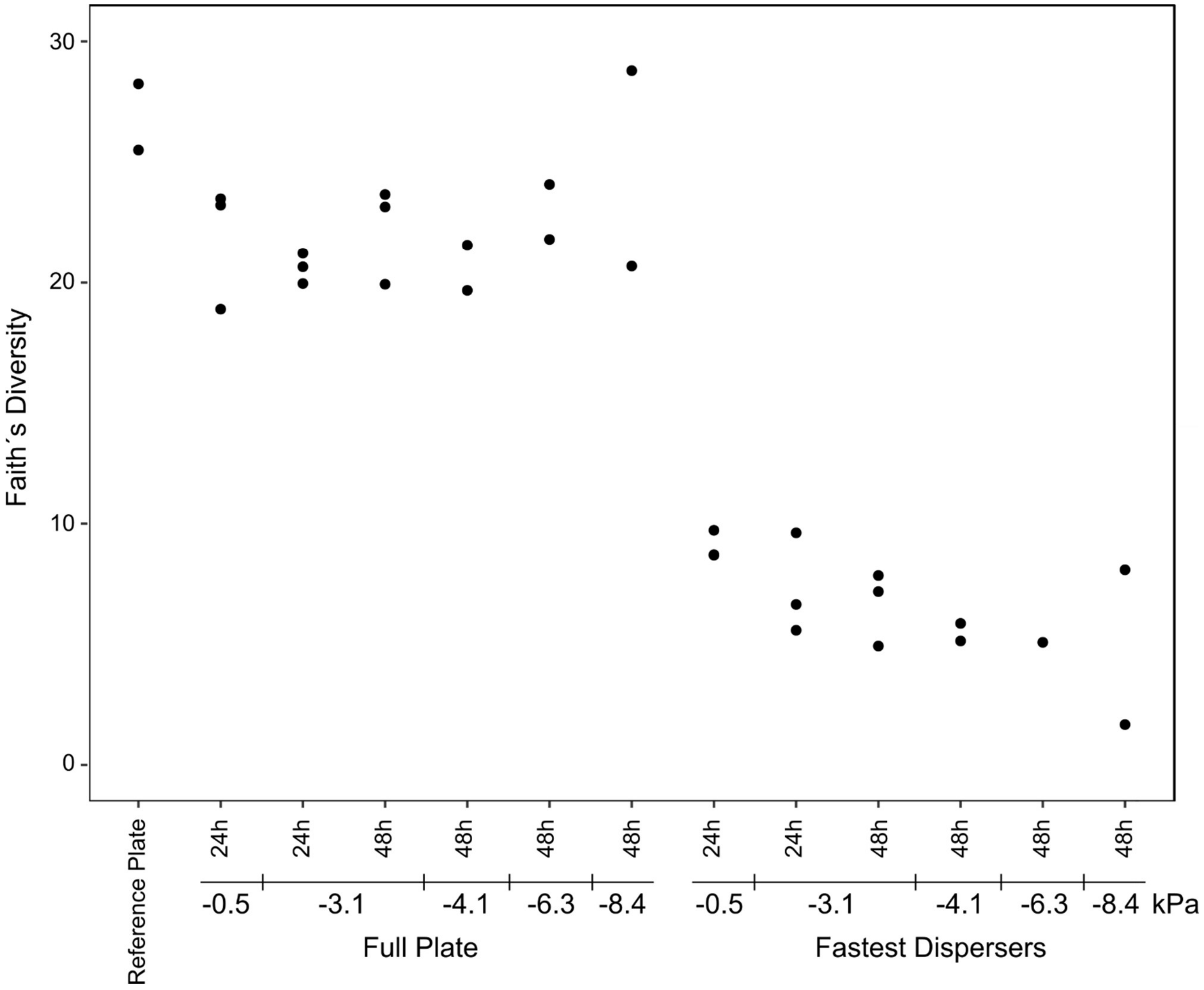

Figure S14. Estimates of alpha diversity (Faith's phylogenetic diversity index) for communities derived from plow layer soil samples and incubated at matric potentials from -0.5 kPa to -8.4 kPa for 24 h or 48 h. For each replicate PSM, the total community recovered from the full agar plate (full plate) and the dispersed community is presented. A motility-restricted control (reference plate) is also included. Replicates are depicted as separate dots. The Faith's phylogenetic diversity indices reported are the averages of values obtained for 10 random rarefactions.

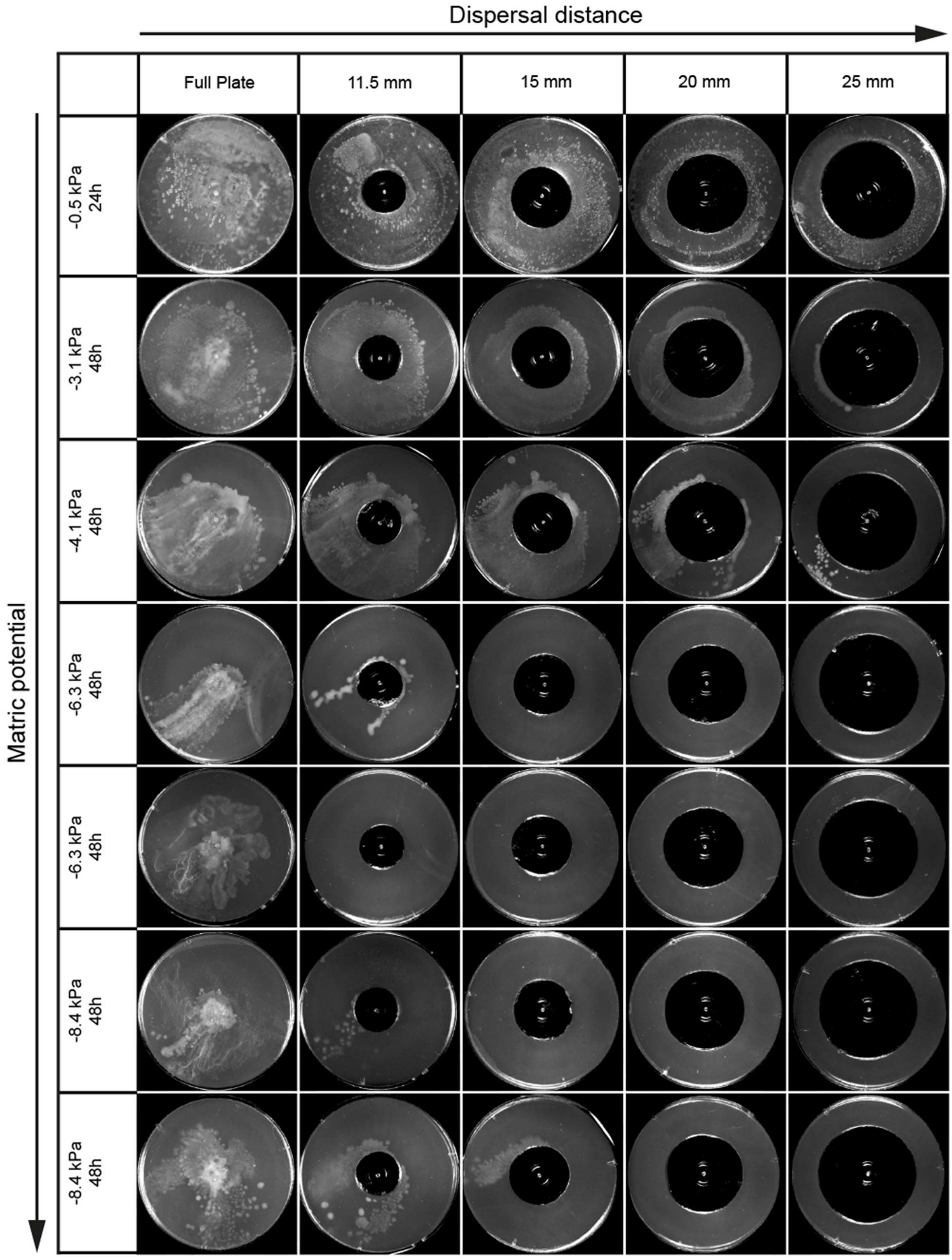
Dispersal distance
Full Plate
11.5 mm
15 mm
20 mm
25 mm
Matric potential
-0.5 kPa 24h
-3.1 kPa 48h
-4.1 kPa 48h
-6.3 kPa 48h
-6.3 kPa 48h
-8.4 kPa 48h
-8.4 kPa 48h

Figure S15. Agar plate coverage by bacterial communities derived from plow layer soil samples exposed to matric potentials from -0.5 kPa to -8.4 kPa for 24 h or 48 h. Sampling was done from “left to right” by pressing the agar plate with the largest hole 25 mm, then 20, 15, 11.5 mm and finishing with a full plate covering the whole surface of the ceramic disc. Agar plates were incubated for 72 h before coverage was evaluated.
